# The Boltzmann distributions of molecular structures predict likely changes through random mutations

**DOI:** 10.1016/j.bpj.2023.10.024

**Published:** 2023-10-29

**Authors:** Nora S. Martin, Sebastian E. Ahnert

**Affiliations:** 1 Rudolf Peierls Centre for Theoretical Physics, University of Oxford, Oxford, United Kingdom; 2 Theory of Condensed Matter Group, Cavendish Laboratory, University of Cambridge, Cambridge, United Kingdom; 3 Sainsbury Laboratory, University of Cambridge, Cambridge, United Kingdom; 4 Department of Chemical Engineering and Biotechnology, University of Cambridge, Cambridge, United Kingdom; 5 The Alan Turing Institute, London, United Kingdom

## Abstract

New folded molecular structures can only evolve after arising through mutations. This aspect is modeled using genotype-phenotype maps, which connect sequence changes through mutations to changes in molecular structures. Previous work has shown that the likelihood of appearing through mutations can differ by orders of magnitude from structure to structure and that this can affect the outcomes of evolutionary processes. Thus, we focus on the phenotypic mutation probabilities $\varphi_{qp}$, i.e., the likelihood that a random mutation changes structure *p* into structure *q*. For both RNA secondary structures and the HP protein model, we show that a simple biophysical principle can explain and predict how this likelihood depends on the new structure *q*: $\varphi_{qp}$ is high if sequences that fold into *p* as the minimum-free-energy structure are likely to have *q* as an alternative structure with high Boltzmann frequency. This generalizes the existing concept of plastogenetic congruence from individual sequences to the entire neutral spaces of structures. Our result helps us understand why some structural changes are more likely than others, may be useful for estimating these likelihoods via sampling and makes a connection to alternative structures with high Boltzmann frequency, which could be relevant in evolutionary processes.

## Significance

The likelihood that random mutations generate a given phenotypic change is important in evolutionary processes. This mutational likelihood can be many orders of magnitude higher for one phenotypic change than for another. Here, we focus on RNA and protein structures and show that these differences are rooted in the biophysics of molecular folding: the likelihood of a mutational change from *p* to *q* is high if sequences that fold into *p* as their energetically optimal fold are likely to have *q* as a suboptimal fold. This result generalizes existing work on the relationship between folding energetics and mutations and helps us understand why some structural changes occur more commonly through mutations than others.

## Introduction

**Figure 1:**
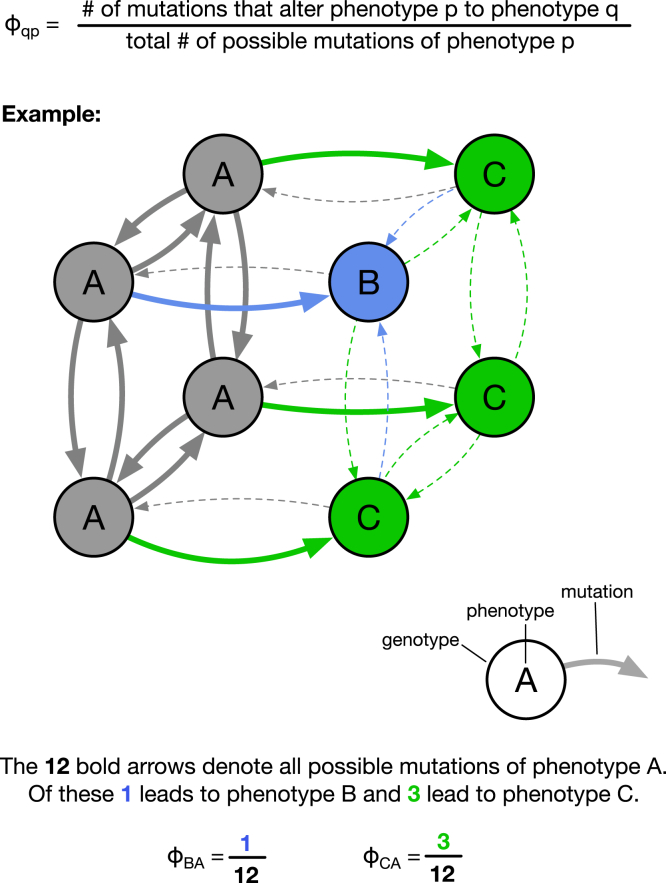
Mutation probability $\varphi_{qp}$. $\varphi_{qp}$ is the fraction of all mutations from an initial phenotype (A, *gray*) to a new phenotype (B or C). This shows a small neutral set with three mutations per genotype. In general, if the initial neutral set contains *x* sequences and there are *y* single-nucleotide substitutions per sequence, then $\varphi_{qp}$ is computed relative to the x×y substitutions that begin from the initial neutral set. For RNA, there are four possible nucleotides per site, so we have y=3L for a sequence of length *L*, whereas the HP protein model only has an alphabet size of two, so we have y=L. To see this figure in color, go online.

**Figure 2:**
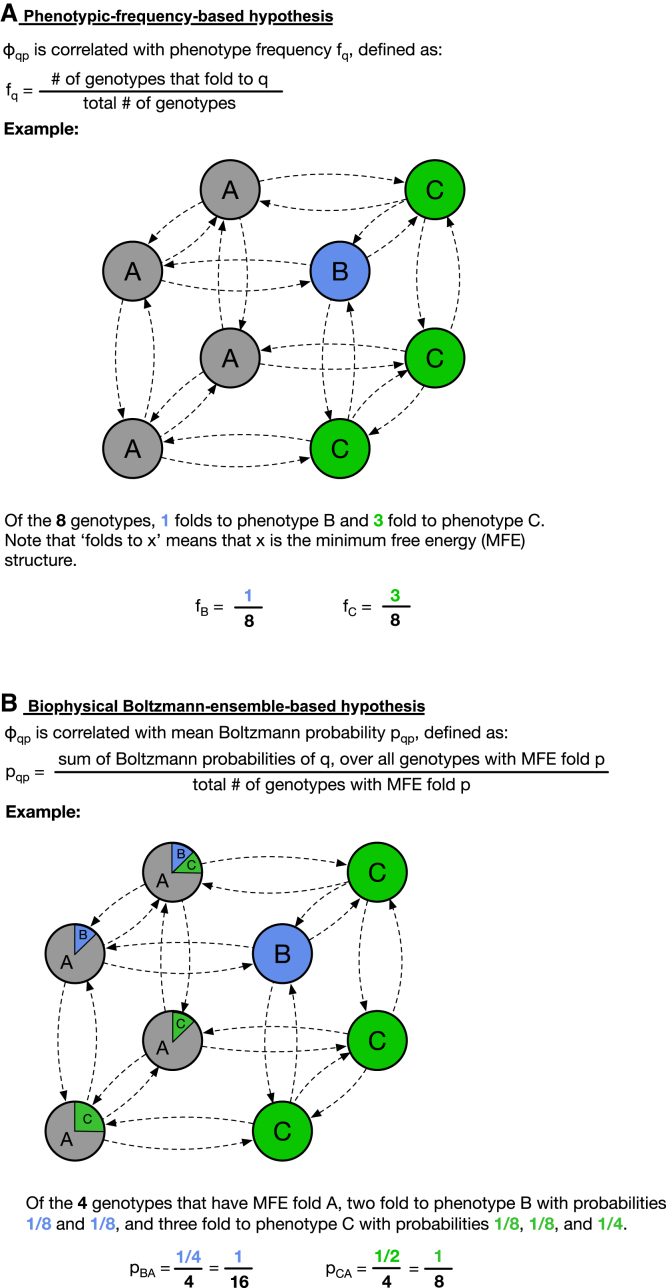
Schematic of the two hypotheses. Phenotypic-frequency-based hypothesis: a good indicator of $\varphi_{qp}$ is the phenotypic frequency of *q*, the total fraction of genotypes that give *q* across the GP map. Biophysical hypothesis: a better indicator of $\varphi_{qp}$ is the mean Boltzmann frequency of structure *q*, where the mean is computed over all genotypes that primarily fold into *p*. To see this figure in color, go online.

For a new molecular structure to evolve, it first has to appear through random mutations. This is not just a qualitative statement but also a quantitative one: if a specific structure appears sooner and more frequently in an evolutionary process, it has a higher chance of going into fixation within a given time frame (1,2,3). This theoretical argument is supported by evolved RNA structures in databases, which tend to be structures that are predicted to appear frequently through random mutations (2,4,5). Thus, the likelihood that a given structure will arise through random mutations is important for evolutionary processes. The quantitative nature of variation can be modeled using a sequence-structure, or genotype-phenotype (GP), map, where random sequence mutations can be mapped to structural changes (6). Due to the huge number of possible sequences (i.e., genotypes) and structures (i.e., phenotypes), this map is best studied computationally, for example using the ViennaRNA package for RNA folding (7) and the HP (‘hydrophobic/polar’) lattice model for protein folding (8). A computational GP map allows us to formalize the notion of “likely” and “unlikely” mutational effects. One central definition for this purpose is the phenotypic mutation probability $\varphi_{qp}$ (1,9) (see also (2,10) but with different notation), which quantifies the likelihood of a specific structural change from an initial structure *p* to a new structure *q*. It is defined as the probability that a mutation on an arbitrary sequence that folds into *p* will lead to a structural change to *q* (9) (see Fig. 1). This mutation probability is useful for predicting structural changes in a population that initially evolves neutrally (1), i.e., maintains structure *p* and remains on the “neutral set” of *p*, which is the set of sequences folding into *p*. Phases of evolution on a neutral set are the norm in evolutionary models on GP maps (see, for example, (1,10,11,12,13)) and apply to a range of scenarios (1): a population can consist of a single sequence at any given time and can move through the neutral set of *p* through genetic drift, or it may consist of many different sequences from the neutral set of *p* at any given time. To predict when these neutral phases will be terminated by the appearance and subsequent fixation of a fitter phenotype *q*, we need the phenotypic mutation probabilities $\varphi_{qp}$ (1). It has been found that the phenotypic mutation probabilities for different structural changes can differ by several orders of magnitude (1,2,11), which means that differences in mutation probabilities can have a big effect on our predictions about evolutionary outcomes. Given that differences in phenotypic mutation probability $\varphi_{qp}$ shape our predictions about evolutionary outcomes, we need to understand which structural changes have high mutation probabilities and why. One approach is to simply identify patterns in the data from a specific GP map model or from a database (11,14), for example by showing that $\varphi_{qp}$ tends to be high for RNA secondary structures if *q* can be obtained from *p* by dissolving a stacked region (11). However, these methods only identify patterns in the data for one specific model—in this case, RNA secondary structures. This shortcoming is addressed by two general approaches. First, it has been argued that the mutation probability $\varphi_{qp}$ from *p* to *q* is proportional to the phenotypic frequency $f_{q}$, which is the probability that an arbitrary sequence folds into *q* (1,9) (as illustrated schematically in Fig. 2 *A*). However, this only works if the initial phenotype *p* has a high phenotypic frequency (9), and even for the highest-frequency phenotype, the correlation was only found to be moderate in the HP protein model (9) (Spearman coefficient ρ≈0.5). Thus, a second approach has recently been proposed based on information-theoretic arguments: this postulates that an upper bound on $\varphi_{qp}$ values can be deduced based on the conditional complexity of *q* given *p* (15), implying that transitions between highly similar structures may be more likely. However, this can only give an upper bound and has not been tested widely. Here, we use a biophysical perspective to understand $\varphi_{qp}$ differences: in the GP map, each sequence is assumed to fold into a single structure, its minimum-free-energy (mfe) structure. However, other suboptimal structures can exist in addition to that structure (16), and these form the Boltzmann ensemble of that sequence. A principle termed plastogenetic congruence postulates that the suboptimal structures in this Boltzmann ensemble can indicate for a specific sequence which structural changes are likely after mutations (17). This has been shown not only for RNA (17) but also for lattice proteins (18). However, this principle is formulated on the level of sequences, but $\varphi_{qp}$ is defined on the level of structures, and thus neutral sets, and it is known that both the effects of mutations (12) and the set of energetically low-lying suboptimal structures (19) differ markedly from sequence to sequence in a neutral set. Nevertheless, there could be a version of plastogenetic congruence that holds on the level of structures, as conjectured in (17): if sequences in the neutral set of *p* tend to have *q* as a suboptimal structure with high Boltzmann frequency, then the phenotypic mutation probability $\varphi_{qp}$ from *p* to *q* is high (see Fig. 2 *B*). Our recent work on insertion/deletion mutations in RNA (20) suggests that this principle holds at least for one specific GP map model using a coarse-grained representation of RNA structures as a phenotype. However, more systematic tests on further models are needed to support this principle and, importantly, to compare it to the phenotypic-frequency-based hypothesis, as sketched in Fig. 2. Thus, the goal is to investigate which of the two different quantities is the best predictor of mutation probabilities $\varphi_{qp}$: the first hypothesis, from (1,9), is that $\varphi_{qp}$ correlates closely with the phenotypic frequency $f_{q}$ of *q*, which is the probability that an arbitrary sequence folds into *q*. The second and biophysical hypothesis, based on the principle of plastogenetic congruence (17) and the data on insertion/deletion mutations in RNA (20), is that $\varphi_{qp}$ values are closely linked to a biophysical quantity, which we denote $p_{qp}$. This $p_{qp}$ is defined as the mean Boltzmann frequency of *q* for a sequence that has *p* as its mfe structure and is thus a measure of how likely temporary switches to *q* are to occur without the presence of mutations. Here, we provide such a systematic comparison for two classic molecular GP map models, RNA secondary structures and the HP protein model. We find that the biophysical Boltzmann-ensemble-based principle reflects $\varphi_{qp}$ more clearly and should therefore be used as a way of understanding why some structural changes are much more likely to occur through mutations than others.

## Materials and methods

### RNA structure predictions

We use the ViennaRNA (7,16,21) package (v.2.4.14, default folding temperature of $T=37^{o}C$, no isolated basepairs) for all structure predictions to ensure consistency: while most thermodynamic structure prediction algorithms are based on the same empirically derived parameter set (21), they usually deviate from the original formulation in small ways (for example, the treatment of all-C loops is simplified in ViennaRNA (7)). We consider structures at the most fine-grained level, where two structures are the same only if they have exactly the same dot-bracket structure, i.e., the same basepairs in the same position (or both have no basepairs). This is the established convention in the field (1,2,5,6,9,10,11,12,17,18,19,22,23,24,25,26) and constitutes the most general way of setting up the sequence-structure map: by picking an appropriate fitness function (for example in (11)), one can study cases where different structural features are functionally important (or not).

#### Mfe structure prediction for a given sequence

We use RNAfold from the ViennaRNA package (7), but we only consider sequences with unique mfe structures as folding (due to the discrete nature of the energy model, this is not guaranteed for all sequences (26)). In order to check for uniqueness of a computed mfe structure, we ran the RNAsubopt (16) function to obtain the full set of structures for an energy range up to 0.02kcal/mol above the mfe structure: this set contains several structures for “degenerate” sequences with multiple mfe structures but only one structure for sequences with a unique mfe structure since free-energy values are returned in discrete steps of 0.1kcal/mol, and so suboptimal structures begin to appear 0.1kcal/mol above the mfe structure.

#### Boltzmann ensemble prediction for a given sequence

In addition to mfe predictions, our calculations rely on Boltzmann frequencies, i.e., the probability that sequence *s* folds into structure *p*. The Boltzmann frequency is calculated as

(1) $$F_{p}=\frac{\exp\;\left(-G_{p}/\left(k_{B}T\right)\right)}{\sum_{q}\;\exp\;\left(-G_{q}/\left(k_{B}T\right)\right)}.$$

Here $G_{p}$ is the free energy of structure *p* in sequence *s* and the sum is over all structures *q*, which sequence *s* can fold into. $k_{B}T$ only depends on the temperature and is $k_{B}T\approx 0.62\;\text{kcal}/\text{mol}$ for our temperature of $T=37^{o}C$. This means that Boltzmann frequencies decrease quickly with temperature: a structure that is $13\frac{\text{kcal}}{\text{mol}}\approx 21k_{B}T$ higher in free energy than the mfe structure has a probability of $\exp\;\left(-21\right)/\left(\exp\;\left(-21\right)+1\right)\approx 7\times 10^{-10}$ if the sequence folds only into that structure and the mfe structure and is even lower if there are other possible structures. There are two possible approaches for computing Boltzmann frequencies in ViennaRNA:

(1) ViennaRNA can return a random structure drawn from the Boltzmann distribution, and one could infer the probability based on many such draws. However, this quickly becomes inaccurate for higher-free-energy structures since we have seen that Boltzmann frequencies can be very small, which makes them difficult to infer from a sample.
(2) We can identify all low-free-energy (i.e., high-Boltzmann-frequency) structures within a given energy range of the mfe structure using the RNAsubopt (16) function. While also an approximation, this allows us to make guarantees: by including structures up to 15kcal/mol, we can guarantee that structures that are not considered have at most a Boltzmann frequency of $\exp\;\left(-15\;\text{kcal}/\text{mol}/\left(k_{B}T\right)\right)\approx 3\times 10^{-11}$. Thus, we choose this approach.

The reason for choosing a fixed cutoff of 15kcal/mol is that higher cutoffs led to an infeasible memory usage in the further data processing: since we compute the Boltzmann frequencies of >10^6^ sequences, it was infeasible to keep track of structures that might only appear with very low probabilities of $\approx 10^{-11}$ for a single sequence in this sample (there is a high enough number of $\approx 10^{6}$ combinatorically possible structures of length L=30 with no isolated basepairs (27) for this to be an issue). For our calculations, where we are interested in averages of Boltzmann frequencies (see Figs. 2 *B* and 4), this cutoff is appropriate: in the worst case, our cutoff would disregard a structure *s* that falls just above the cutoff for every sequence in our sample. Then, its true average Boltzmann frequency would be around $\approx 3\times 10^{-11}$ but would be zero in our calculations. This would not be a problem since values as low as this would not be included in our further analysis anyway due to the sampling limitations discussed in GP map analyses for RNA. Once we have a list of low-energy structures for a given sequence, we simply compute the Boltzmann frequencies using Eq. (1) (we did not use ViennaRNA’s inbuilt function because we found this to be inaccurate when the isolated basepair setting was switched off when testing our method on short sequences of L=15, where exact calculations with no approximations are feasible).

### GP map analyses for RNA

**Figure 3:**
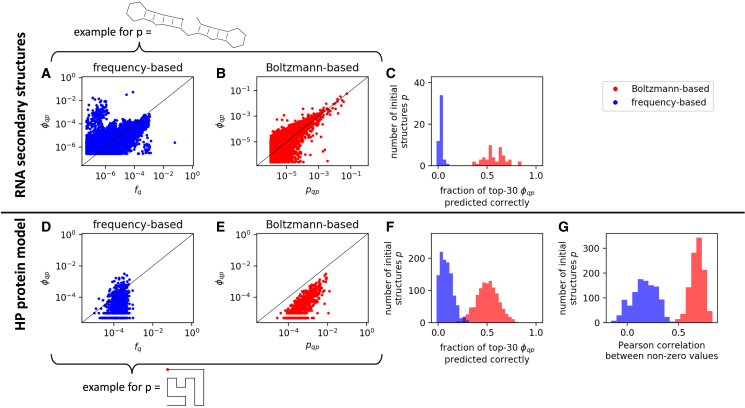
Data for the two hypotheses for the RNA secondary structure GP map (*A–C*) and the HP protein structure GP map (*D–G*). (*A*) Phenotypic-frequency-based hypothesis for one initial RNA structure *p* as an example (*sketched at the top*): the $\varphi_{qp}$ values for mutating from this initial structure *p* to a new structure *q* are plotted against the phenotypic frequency $f_{q}$ of the new structure *q*. (*B*) Biophysical Boltzmann-ensemble-based hypothesis for the same initial RNA structure *p*: the $\varphi_{qp}$ values for this initial structure are plotted against the biophysical quantity $p_{qp}$, i.e., the Boltzmann frequency of the new structure *q* averaged over sequences with mfe structure *p*. The black lines indicate x=y. (*C*) The analysis is repeated for 50 different initial structures *p*. For each *p*, we score each hypothesis by evaluating how many of the structures with the 30 highest $\varphi_{qp}$ values are captured correctly by each hypothesis. (*D–G*) Same analysis for the HP protein model. (*D*) Phenotypic-frequency-based hypothesis for one specific initial structure (*sketched on the bottom*, the *red dot* indicates the start position). (*E*) Biophysical Boltzmann-ensemble-based hypothesis for the same structure. (*F* and *G*) The analysis is repeated for all 1081 HP structures that fold as mfe structures: the biophysical hypothesis scores better, regardless of whether we focus on the top-$\varphi_{qp}$ structures (*F*) or compute the Pearson correlation coefficients on a log-log scale (*G*). To see this figure in color, go online.

**Figure 4:**
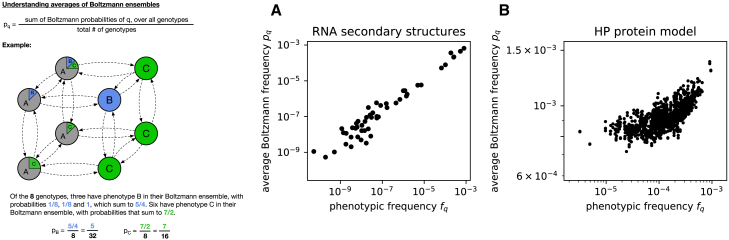
Boltzmann frequencies and phenotypic frequencies. Here, we compare phenotypic frequencies $f_{q}$ to $p_{q}$, the average Boltzmann frequency of structure *q* over all sequences. The definition of $p_{q}$ is illustrated in the schematic on the left. (*A*) The average Boltzmann frequency $p_{q}$ of an RNA secondary structure *q* is plotted against the phenotypic frequency $f_{q}$ of this structure, i.e., the probability that a random sequence folds into *q* as its minimum-free-energy structure. (*B*) Same for the HP protein model. The same structures are shown as in Fig. 3, *C* and *F*; the sets of structures and the sampling methods used to estimate $f_{q}$ and $p_{q}$ are detailed in the materials and methods. To see this figure in color, go online.

In the last sections, we have described how to fold an individual RNA sequence and obtain its mfe structure and Boltzmann ensemble. However, the full GP map for a sequence length of L=30 contains $4^{30}\approx 10^{18}$ sequences. Thus, we have to rely on sampling approaches to estimate properties like $\varphi_{qp}$, $f_{q}$, and $p_{qp}$, as described in the following. $\varphi_{qp}$ and $p_{qp}$ are both defined as averages over the neutral set of a structure *p* and are approximated by two samples of $2\times 10^{5}$ sequences per neutral set (one for $\varphi_{qp}$ estimates and one for $p_{qp}$ estimates). These samples are generated with Weiß and Ahnert’s site-scanning method (25) (parameters: 100 site-scanning processes with $10^{5}$ steps and subsequent subsampling of one in 50 sequences), using a version of this method (22) that includes basepair swaps and is therefore more suitable for sampling from neutral sets, not just their connected components. In the supporting material (section S1.1.1), we provide further details and show that the sample size is large enough to reliably compute $\varphi_{qp}$ and $p_{qp}$ values ⪆ 10^−4^; in the supporting material (section S1.1.2), we further test for systematic bias in the sampling method. Using this sampling method, we computed $\varphi_{qp}$ and $p_{qp}$ for a total of 50 initial structures *p*. These were randomly drawn out of all structures obtained from folding $10^{8}$ sequences—the random draw is weighted such that there are a similar number of structures with *x* stacks. Since the number of stacks is strongly correlated with neutral set sizes (5), which in turn are correlated with robustness (9) and stability (22), this ensures that the chosen structures are qualitatively different. To estimate phenotypic frequencies $f_{q}$ for Fig. 3, we simply fold $10^{8}$ random sequences and approximate the phenotypic frequency of each structure by its frequency in the sample. This is an unbiased estimator of $f_{q}$ but inaccurate for $f_{q}⪅10^{-8}$, and thus we only plot values of $f_{q}\geq 5/10^{-8}$. Similarly, we estimated the Boltzmann averages $p_{q}$ in Fig. 4 by folding $10^{7}$ random sequences (a lower sample size because we get more nuanced quantitative information from each sequence: a Boltzmann ensemble and not just a single structure). Since our 50 selected structures include low-frequency structures (they were selected to represent a range of neutral set sizes), where the requirement $f_{q}\geq 5/10^{-8}$ does not apply, we estimate their phenotypic frequencies for Fig. 4 with the program by Jörg et al. (24) (with isolated basepairs switched off), which uses a nested Markov chain Monte Carlo algorithm in combination with ViennaRNA predictions. This method does not test for the uniqueness of mfe structures, but the impact of this is minimal for $f_{q}$ predictions (22). The predictions for structures with $f_{q}\geq 5/10^{-8}$, where both sampling methods can be used to estimate $f_{q}$ values, are in excellent agreement between the two methods (see Fig. S5).

### HP protein model

Our data rely on a full enumeration of all HP sequences of length L=25 and their folded structures on a compact lattice, using a simple energy model (28) with a stabilising contact energy of one unit for two hydrophobic residues and no energy contribution otherwise. We follow the steps outlined by Greenbury et al. (9,23,29) in the construction of the GP map (including the convention that sequences with multiple mfe structures are considered nonfolding) and test our methods against their data (23). However, in this article, we treat two structures as distinct if they have reversed directionality, i.e., if the structure looks identical except with the N-terminus and C-terminus swapped, as in (30). This convention was chosen for consistency with the RNA folding model, where information on directionality in the folded structure is also retained. The only free parameter in the HP model is the reduced temperature (relative to the HP interaction strength), for which we use $k_{B}T=0.5$ since this represents a realistic middle ground between $k_{B}T=0.1$ on the one extreme, where the protein has no plasticity and typically spends >99% of time in the ground state, and $k_{B}T=1$ on the other extreme, where the ground state accounts for less than 5% of the Boltzmann ensemble of a typical sequence. However, our results also hold for $k_{B}T=0.1$ and 1 (shown in section S7 of the supporting material).

### GP map analyses for the HP protein model

$f_{q}$ and $\varphi_{qp}$ values are computed exactly. $p_{qp}$ values are approximated by the average over $10^{3}$ sequences drawn with replacement from the neutral set of *p*. We show in the supporting material (section S1.2) that this sample is large enough for sampling errors to be negligible. In a similar way, Boltzmann averages over arbitrary sequences ($p_{q}$) are approximated by the average over $10^{5}$ sequences.

## Results

### $\varphi_{qp}$ and Boltzmann frequencies

First, we test both hypotheses in Fig. 2 for one specific RNA structure *p* (shown in Fig. 3, the structure was chosen to have a median neutral set size: it is the 26th-highest neutral set size out of the 50 structures in our dataset): we plot both the phenotypic frequency $f_{q}$ (*blue*, Fig. 3 *A*) and the Boltzmann-ensemble-based biophysical quantity $p_{qp}$ (*red*, Fig. 3 *B*) against the corresponding $\varphi_{qp}$ values. We find that the correlation is much clearer for the biophysical quantity. In order to test if our results generalize beyond the specific RNA structure used in Fig. 3, *A* and *B*, we collected the same data for 50 initial structures *p* (full data shown in Figs. S9–S13). These 50 structures are very diverse, with between one and five stacks (see GP map analyses for RNA and Fig. S6). Since the number of stacks is correlated with neutral set size (5) and thus with other mutational and biophysical quantities (9, 22), this means that we also have a range of mutational robustness and thermodynamic stability values in our dataset. For each initial structure *p*, we evaluate how many of the structures with the 30 highest $\varphi_{qp}$ values are predicted correctly by each of the two hypotheses, based on phenotypic frequencies $f_{q}$ or the biophysical quantity $p_{qp}$. We chose a performance metric based on the highest $\varphi_{qp}$ values for the following reasons: The highest $\varphi_{qp}$ values are of practical importance since they represent the likeliest mutational transitions, and they are insensitive to sampling errors in our calculations: sampling errors become important around $\varphi_{qp}⪅10^{-4}$ (see section S1.1.1), whereas the highest $\varphi_{qp}$ values are typically $\varphi_{qp}⪆10^{-2}$, two orders of magnitude higher, and therefore we can determine the highest values with high certainty. Fig. 3 *C* shows this performance metric for our 50 RNA datasets, each corresponding to one initial structure *p*: we find that, indeed, the biophysical Boltzmann-frequency-based quantity is a better predictor of $\varphi_{qp}$ than phenotypic frequencies. To understand why phenotypic frequencies $f_{q}$ might not be a good predictor, we consider the outliers in Fig. 3 *A* in more detail: some of the most likely mutational changes (i.e., high $\varphi_{qp}$ values) actually correspond to low-$f_{q}$ structures. These transitions, which are locally frequent from *p* (i.e., have a high $\varphi_{qp}$) but globally rare (i.e., have low $f_{q}$), are transitions between two similar structures *p* and *q* (see section S2 and (9,15)). These transitions are likely to play a key role in evolutionary processes since incremental variation is more likely to be adaptive than larger structural changes (see (23) for an example). The biophysical Boltzmann-frequency-based approach in Fig. 3 *B* correctly captures these high-$\varphi_{qp}$ transitions between similar structures. Next, we repeat our analysis for the HP protein model (Fig. 3, *D–G*): since the sequence space is smaller in this model, we use exact data without sampling, except for the biophysical quantity $p_{qp}$. This also means that we have data for all possible initial structures *p* and for all possible structural changes for each *p* (see Figs. S14–S18 for further choices of *p*), and we use all these data in Fig. 3, *F* and *G*. Since sampling errors are not an issue in our HP data, we can use the full range of $\varphi_{qp}$ values and compare the different hypotheses not only based on the highest-$\varphi_{qp}$ structures (Fig. 3 *F*) but also by computing Pearson correlation coefficients (Fig. 3 *G*). We find that, as before for RNA, the biophysical Boltzmann-frequency-based approach is a better indicator of which structural changes are most likely to occur through mutations. To conclude, neutral set averages of Boltzmann frequencies $p_{qp}$ are a good indicator of which structural transitions are likely to occur through mutations. Here, we have shown that they are a better indicator than phenotypic frequencies since this is the most well-known approximation for $\varphi_{qp}$, but in the supporting material (section S4), we also show that they outperform other ways in which $\varphi_{qp}$ might be estimated, for example based on structural similarity or by using the upper bound given by the conditional complexity arguments from Dingle et al. (15).

### Boltzmann frequencies and phenotypic frequencies

To better understand the neutral set averages of Boltzmann frequencies $p_{qp}$, which are at the center of our analysis, we also evaluated a closely related quantity, namely the averages of Boltzmann frequencies over random sequences. The data in Fig. 4 indicate that this quantity $p_{q}$, the mean Boltzmann frequency of structure *q* over all sequences in the GP map, is correlated with the phenotypic frequency of that structure, especially in the RNA map. This holds regardless of whether mfe structures are included in the averages, as in Fig. 4, or not, as shown in Fig. S28. This result agrees with existing data for RNA (4,31). It is also consistent with theoretical arguments (32) for a Boltzmann-like trend in phenotypic frequencies, where energetically unfavorable features are associated with an exponential decrease in phenotypic frequencies. Not all assumptions behind this theoretical claim are met in our case (for example, that the mean free energy of folding for a random sequence differs from structure to structure, which is not true in many compact HP models (33), and the assumption of constant sequence composition in the random energy model (34)). The fact that not even the key assumption on free-energy distributions holds for the compact HP model may partially explain why the correlation is less clear for the HP model. We can apply the insight from Fig. 4 to neutral set averages as follows: the sequences in a large neutral set are (almost) as diverse as arbitrary sequences, especially for RNA (section S6). Therefore, it is likely that the neutral set averages of Boltzmann frequencies $p_{qp}$ in large neutral sets are similar to the averages over arbitrary sequences $p_{q}$. This would mean that for structures *p* with large neutral sets, we have $p_{qp}\propto f_{q}$. Thus, we recover the existing hypothesis (9) that for the special case of an initial structure with a large neutral set, there is a high correlation between $\varphi_{qp}$ and the phenotypic frequency of the new structure $f_{q}$.

## Discussion

In this article, we have shown that averages over Boltzmann ensembles capture two key GP map quantities: first, if sequences folding into an initial structure *p* have a structure *q* as a high-Boltzmann-frequency alternative, then there are many mutations that transform *p* into *q*, as previously shown in our data from the RNAshapes model (20). This argument is a better indicator of likely mutational transitions than simply the phenotypic frequency $f_{q}$ of structure *q*. Our results thus show that the existing principle of plastogenetic congruence (17) can be generalized from individual sequences to averages over the neutral set of structures, even though the mutational neighborhood (9,12) and the ensemble of suboptimal structures with high Boltzmann frequencies (19) differs markedly from sequence to sequence in a neutral set. Secondly, we found that structures that have high Boltzmann probabilities in arbitrary sequences also have high phenotypic frequencies $f_{q}$, in agreement with previous data for RNA (4,31). Intuitively, plastogenetic congruence should apply for well-behaved energy functions, as in the theoretical calculations in (35), where free energies vary by a small amount after a single mutation. Under these conditions, the mfe structure after a mutation already has to exist as a low-energy structure before the mutation, and so we have plastogenetic congruence. The HP model has such a well-behaved energy function because each residue has up to three contacts, and so the free energy of a given structure can only change by up to three units after a mutation. For RNA structures, however, some mutations will cause free-energy jumps in some structures by preventing a basepair from forming and thus making it impossible for the structure to fold at all. Despite this potential caveat, existing work on plastogenetic congruence (17) in RNA, as well as the data in this article, demonstrates that the concept still holds for RNA. In fact, one classic paper (17) uses basepairing constraints to argue why rare mutational transitions might also be rare plastic transitions. Our results show that the concept holds for both the HP model and the RNA model, indicating that the free-energy jumps caused by basepairing constraints neither prevent nor enable plastogenetic congruence and its generalization to neutral sets. The fact that plastogenetic congruence and similar effects are found in a range of contexts (for example, (36,37,38,39,40,41)) indicates that our results may also hold more broadly for a range of GP maps. However, many of these analyses focus on continuous phenotypic and structural changes, whereas here, we have focused specifically on the probabilities of obtaining specific discrete structural changes because these probabilities have been shown to be highly biased (11) and impact evolutionary processes (1). Future work should investigate connections between these different perspectives on mutational changes. One implication of our results is that structures that have high average Boltzmann probabilities and are thus likely to evolve as suboptimal structures also have high $\varphi_{qp}$ values and are thus more likely to evolve as mfe structures, as conjectured in Ancel and Fontana’s original paper on plastogenetic congruence (17). This applies for arbitrary sequences, where the relevant quantities are $f_{q}$ and $p_{q}$, and for a given initial structure, where the relevant quantities are $\varphi_{qp}$ and $p_{qp}$. This finding is highly relevant in cases where the fitness of a sequence is not determined simply by the identity of its mfe structure but instead depends on the Boltzmann frequency of the correctly folded structure, as was found in a large-scale experimental study on tRNAs (42). In this case, the likelihood of evolving a given structure *q* would not be given by $\varphi_{qp}$ and $f_{q}$ values, as traditionally assumed in models (1,5,11,15,43), but by a quantity that reflects the possibility that *q* could emerge as a suboptimal structure, i.e., a quantity related to the Boltzmann averages $p_{q}$ or $p_{qp}$. Given the progress in calculating the probabilities and timescales on which different structures evolve in the simpler models where only mfe structures matter (1,2,3), future work should investigate these questions systematically for cases in which the fitness depends on suboptimal structures. In this case, the established definition of $\varphi_{qp}$ should be adjusted to include these suboptimal structures of each sequence, similar to the rates in (44). Our data on the parallels between Boltzmann ensembles and mutational changes can guide such analyses. Furthermore, our results could have a practical application in sampling methods: usually, GP map analyses are restricted to short sequences because of the computational cost required to sample a sufficient number of sequences to estimate quantities like phenotypic frequencies and mutation probabilities $\varphi_{qp}$. Sophisticated methods that reduce the required sample sizes are therefore important (24,25). Relying on the biophysical quantity $p_{qp}$ could be one option of reducing sampling sizes when estimating $\varphi_{qp}$ data: if $\varphi_{qp}$ was estimated directly from a sequence sample, one needs to fold all the sequences in the sample and their mutational neighbors, but for the biophysical quantity $p_{qp}$, one only needs to obtain the Boltzmann ensemble of the sequences itself. This is especially useful in cases like the HP model, where entire Boltzmann ensembles can be predicted as quickly as the mfe structure since the mfe structure is usually identified by computing the energy of all folds (for example, (8,29)). It may even be possible to develop methods that estimate $p_{qp}$ without extensive sampling by building on existing techniques (45,46) developed for bistable sequences, i.e., sequences that fold into both *p* and *q* with high probabilities. Potential avenues for future research in this direction are discussed in more detail in section S8 of the supporting material. In a similar way, the results may be useful when making inferences from partial experimental data on mutational changes and fluctuations.

## Data and code availability

The code behind this analysis can be found at https://github.com/noramartin/mutation_probabilities.

## Author contributions

N.S.M. designed and performed the computational analysis and wrote the manuscript. S.E.A. supervised the project.

## Supporting citations

References (47,48,49,50,51,52,53,54,55,56,57,58,59) appear in the Supporting Material.

## Supporting material

Document S1. Supporting material and Figures S1–S32

Document S2. Article plus supporting material
