# Supplementary material for: The Boltzmann distributions of molecular structures predict likely changes through random mutations
Source: Biophys J. 2023 Oct 29;122(22):4467–75. doi: 10.1016/j.bpj.2023.10.024 (PMC10698324; doi:10.1016/j.bpj.2023.10.024)
Supplement: Document S2. Article plus supporting material [file mmc2.pdf]

Biophysical Journal
Article

Biophysical Society

The Boltzmann distributions of molecular structures predict likely changes through random mutations

Nora S. Martin[1,2,3,*] and Sebastian E. Ahnert[4,5]
[1]Rudolf Peierls Centre for Theoretical Physics, University of Oxford, Oxford, United Kingdom; [2]Theory of Condensed Matter Group, Cavendish Laboratory, University of Cambridge, Cambridge, United Kingdom; [3]Sainsbury Laboratory, University of Cambridge, Cambridge, United Kingdom; [4]Department of Chemical Engineering and Biotechnology, University of Cambridge, Cambridge, United Kingdom; and [5]The Alan Turing Institute, London, United Kingdom

ABSTRACT New folded molecular structures can only evolve after arising through mutations. This aspect is modeled using genotype-phenotype maps, which connect sequence changes through mutations to changes in molecular structures. Previous work has shown that the likelihood of appearing through mutations can differ by orders of magnitude from structure to structure and that this can affect the outcomes of evolutionary processes. Thus, we focus on the phenotypic mutation probabilities φ_{qp}, i.e., the likelihood that a random mutation changes structure p into structure q. For both RNA secondary structures and the HP protein model, we show that a simple biophysical principle can explain and predict how this likelihood depends on the new structure q: φ_{qp} is high if sequences that fold into p as the minimum-free-energy structure are likely to have q as an alternative structure with high Boltzmann frequency. This generalizes the existing concept of plastogenetic congruence from individual sequences to the entire neutral spaces of structures. Our result helps us understand why some structural changes are more likely than others, may be useful for estimating these likelihoods via sampling and makes a connection to alternative structures with high Boltzmann frequency, which could be relevant in evolutionary processes.

SIGNIFICANCE The likelihood that random mutations generate a given phenotypic change is important in evolutionary processes. This mutational likelihood can be many orders of magnitude higher for one phenotypic change than for another. Here, we focus on RNA and protein structures and show that these differences are rooted in the biophysics of molecular folding: the likelihood of a mutational change from p to q is high if sequences that fold into p as their energetically optimal fold are likely to have q as a suboptimal fold. This result generalizes existing work on the relationship between folding energetics and mutations and helps us understand why some structural changes occur more commonly through mutations than others.

INTRODUCTION

For a new molecular structure to evolve, it first has to appear through random mutations. This is not just a qualitative statement but also a quantitative one: if a specific structure appears sooner and more frequently in an evolutionary process, it has a higher chance of going into fixation within a given time frame (1–3). This theoretical argument is supported by evolved RNA structures in databases, which tend to be structures that are predicted to appear frequently through random mutations (2,4,5). Thus, the likelihood that a given structure will arise through random mutations is important for evolutionary processes. The quantitative nature of variation can be modeled using a sequence-structure, or genotype-phenotype (GP), map, where random sequence mutations can be mapped to structural changes (6). Due to the huge number of possible sequences (i.e., genotypes) and structures (i.e., phenotypes), this map is best studied computationally, for example using the ViennaRNA package for RNA folding (7) and the HP ('hydrophobic/polar') lattice model for protein folding (8). A computational GP map allows us to formalize the notion of "likely" and "unlikely" mutational effects. One central definition for this purpose is the phenotypic mutation probability φ_{qp} (1,9) (see also (2,10) but with different notation), which quantifies

Submitted April 14, 2023, and accepted for publication October 20, 2023.

*Correspondence: nora.martin@crg.eu

Nora S. Martin's present address is CRG (Barcelona Collaboratorium for Modeling and Predictive Biology), Barcelona Institute of Science and Technology, Barcelona, Spain

Editor: Margaret Shun Cheung.

https://doi.org/10.1016/j.bpj.2023.10.024

© 2023 Biophysical Society.

This is an open access article under the CC BY license (http://creativecommons.org/licenses/by/4.0/).

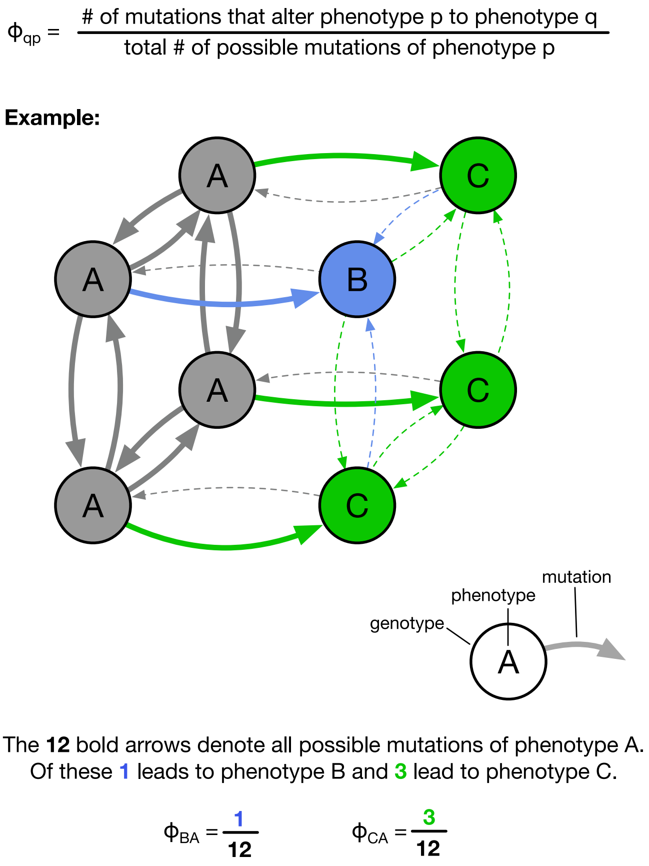

FIGURE 1 Mutation probability φ_{qp}. φ_{qp} is the fraction of all mutations from an initial phenotype (A, *gray*) to a new phenotype (B or C). This shows a small neutral set with three mutations per genotype. In general, if the initial neutral set contains x sequences and there are y single-nucleotide substitutions per sequence, then φ_{qp} is computed relative to the $x \times y$ substitutions that begin from the initial neutral set. For RNA, there are four possible nucleotides per site, so we have $y = 3L$ for a sequence of length L, whereas the HP protein model only has an alphabet size of two, so we have $y = L$. To see this figure in color, go online.

the likelihood of a specific structural change from an initial structure p to a new structure q. It is defined as the probability that a mutation on an arbitrary sequence that folds into p will lead to a structural change to q (9) (see Fig. 1). This mutation probability is useful for predicting structural changes in a population that initially evolves neutrally (1), i.e., maintains structure p and remains on the "neutral set" of p, which is the set of sequences folding into p. Phases of evolution on a neutral set are the norm in evolutionary models on GP maps (see, for example, (1,10–13)) and apply to a range of scenarios (1): a population can consist of a single sequence at any given time and can move through the neutral set of p through genetic drift, or it may consist of many different sequences from the neutral set of p at any given time. To predict when these neutral phases will be terminated by the appearance and subsequent fixation of a fitter phenotype q, we need the phenotypic mutation probabilities φ_{qp} (1). It has been found that the phenotypic mutation probabilities for different structural changes can differ by several orders of magnitude (1,2,11), which means that differences in mutation probabilities can have a big effect on our predictions about evolutionary outcomes. Given that differences in phenotypic mutation probability φ_{qp} shape our predictions about evolutionary outcomes, we need to understand which structural changes have high mutation probabilities and why. One approach is to simply identify patterns in the data from a specific GP map model or from a database (11,14), for example by showing that φ_{qp} tends to be high for RNA secondary structures if q can be obtained from p by dissolving a stacked region (11). However, these methods only identify patterns in the data for one specific model—in this case, RNA secondary structures. This shortcoming is addressed by two general approaches. First, it has been argued that the mutation probability φ_{qp} from p to q is proportional to the phenotypic frequency f_q, which is the probability that an arbitrary sequence folds into q (1,9) (as illustrated schematically in Fig. 2 *A*). However, this only works if the initial phenotype p has a high phenotypic frequency (9), and even for the highest-frequency phenotype, the correlation was only found to be moderate in the HP protein model (9) (Spearman coefficient $\rho \approx 0.5$). Thus, a second approach has recently been proposed based on information-theoretic arguments: this postulates that an upper bound on φ_{qp} values can be deduced based on the conditional complexity of q given p (15), implying that transitions between highly similar structures may be more likely. However, this can only give an upper bound and has not been tested widely. Here, we use a biophysical perspective to understand φ_{qp} differences: in the GP map, each sequence is assumed to fold into a single structure, its minimum-free-energy (mfe) structure. However, other suboptimal structures can exist in addition to that structure (16), and these form the Boltzmann ensemble of that sequence. A principle termed plastogenetic congruence postulates that the suboptimal structures in this Boltzmann ensemble can indicate for a specific sequence which structural changes are likely after mutations (17). This has been shown not only for RNA (17) but also for lattice proteins (18). However, this principle is formulated on the level of sequences, but φ_{qp} is defined on the level of structures, and thus neutral sets, and it is known that both the effects of mutations (12) and the set of energetically low-lying suboptimal structures (19) differ markedly from sequence to sequence in a neutral set. Nevertheless, there could be a version of plastogenetic congruence that holds on the level of structures, as conjectured in (17): if sequences in the neutral set of p tend to have q as a suboptimal structure with high Boltzmann frequency, then the phenotypic mutation probability φ_{qp} from p to q is high (see Fig. 2 *B*). Our recent work on insertion/deletion mutations in RNA (20) suggests that this principle holds at least for one specific GP map model using a coarse-grained representation of RNA structures as a phenotype. However, more systematic tests on further models are needed to support this principle

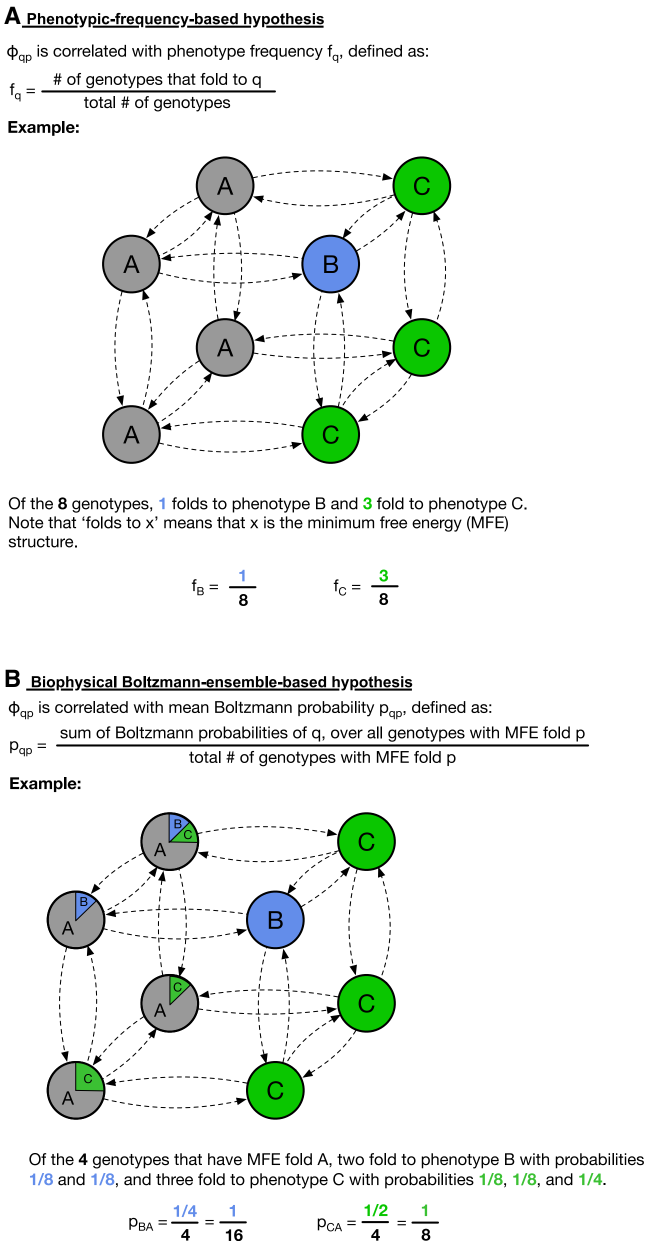

FIGURE 2 Schematic of the two hypotheses. Phenotypic-frequency-based hypothesis: a good indicator of φ_{qp} is the phenotypic frequency of q, the total fraction of genotypes that give q across the GP map. Biophysical hypothesis: a better indicator of φ_{qp} is the mean Boltzmann frequency of structure q, where the mean is computed over all genotypes that primarily fold into p. To see this figure in color, go online.

and, importantly, to compare it to the phenotypic-frequency-based hypothesis, as sketched in Fig. 2. Thus, the goal is to investigate which of the two different quantities is the best predictor of mutation probabilities φ_{qp}: the first hypothesis, from (1,9), is that φ_{qp} correlates closely with the phenotypic frequency f_q of q, which is the probability that an arbitrary sequence folds into q. The second and biophysical hypothesis, based on the principle of plastogenetic congruence (17) and the data on insertion/deletion mutations in RNA (20), is that φ_{qp} values are closely linked to a biophysical quantity, which we denote p_{qp}. This p_{qp} is defined as the mean Boltzmann frequency of q for a sequence that has p as its mfe structure and is thus a measure of how likely temporary switches to q are to occur without the presence of mutations. Here, we provide such a systematic comparison for two classic molecular GP map models, RNA secondary structures and the HP protein model. We find that the biophysical Boltzmann-ensemble-based principle reflects φ_{qp} more clearly and should therefore be used as a way of understanding why some structural changes are much more likely to occur through mutations than others.

MATERIALS AND METHODS

RNA structure predictions

We use the ViennaRNA (7,16,21) package (v.2.4.14, default folding temperature of $T = 37^{\circ}$C, no isolated basepairs) for all structure predictions to ensure consistency: while most thermodynamic structure prediction algorithms are based on the same empirically derived parameter set (21), they usually deviate from the original formulation in small ways (for example, the treatment of all-C loops is simplified in ViennaRNA (7)). We consider structures at the most fine-grained level, where two structures are the same only if they have exactly the same dot-bracket structure, i.e., the same basepairs in the same position (or both have no basepairs). This is the established convention in the field (1,2,5,6,9–12,17–19,22–26) and constitutes the most general way of setting up the sequence-structure map: by picking an appropriate fitness function (for example in (11)), one can study cases where different structural features are functionally important (or not).

Mfe structure prediction for a given sequence

We use RNAfold from the ViennaRNA package (7), but we only consider sequences with unique mfe structures as folding (due to the discrete nature of the energy model, this is not guaranteed for all sequences (26)). In order to check for uniqueness of a computed mfe structure, we ran the RNAsubopt (16) function to obtain the full set of structures for an energy range up to 0.02 kcal/mol above the mfe structure: this set contains several structures for "degenerate" sequences with multiple mfe structures but only one structure for sequences with a unique mfe structure since free-energy values are returned in discrete steps of 0.1 kcal/mol, and so suboptimal structures begin to appear 0.1 kcal/mol above the mfe structure.

Boltzmann ensemble prediction for a given sequence

In addition to mfe predictions, our calculations rely on Boltzmann frequencies, i.e., the probability that sequence s folds into structure p. The Boltzmann frequency is calculated as

$$F_p = \frac{\exp\left(-G_p/(k_BT)\right)}{\sum_q \exp\left(-G_q/(k_BT)\right)}. \quad (1)$$

Here G_p is the free energy of structure p in sequence s and the sum is over all structures q, which sequence s can fold into. k_BT only depends on the temperature and is $k_BT \approx 0.62$ kcal/mol for our temperature of $T = 37^{\circ}$C. This means that Boltzmann frequencies decrease quickly with temperature: a structure that is $13\frac{\text{kcal}}{\text{mol}} \approx 21 k_BT$ higher in free energy than the mfe structure has a probability of $\exp(-21)/(\exp(-21)+1) \approx 7\times10^{-10}$ if the sequence folds only into that structure and the mfe structure and is even lower if there are other possible structures. There are two possible approaches for computing Boltzmann frequencies in ViennaRNA:

(1) ViennaRNA can return a random structure drawn from the Boltzmann distribution, and one could infer the probability based on many such draws. However, this quickly becomes inaccurate for higher-free-energy structures since we have seen that Boltzmann frequencies can be very small, which makes them difficult to infer from a sample.
(2) We can identify all low-free-energy (i.e., high-Boltzmann-frequency) structures within a given energy range of the mfe structure using the RNAsubopt (16) function. While also an approximation, this allows us to make guarantees: by including structures up to 15 kcal/ mol, we can guarantee that structures that are not considered have at most a Boltzmann frequency of $\exp(-\ 15\ \text{kcal}/\text{mol}/(k_BT)) \approx 3 \times 10^{-11}$. Thus, we choose this approach.

The reason for choosing a fixed cutoff of 15 kcal/mol is that higher cutoffs led to an infeasible memory usage in the further data processing: since we compute the Boltzmann frequencies of $>10^6$ sequences, it was infeasible to keep track of structures that might only appear with very low probabilities of $\approx 10^{-11}$ for a single sequence in this sample (there is a high enough number of $\approx 10^6$ combinatorically possible structures of length $L = 30$ with no isolated basepairs (27) for this to be an issue). For our calculations, where we are interested in averages of Boltzmann frequencies (see Figs. 2 *B* and 4), this cutoff is appropriate: in the worst case, our cutoff would disregard a structure s that falls just above the cutoff for every sequence in our sample. Then, its true average Boltzmann frequency would be around $\approx 3 \times 10^{-11}$ but would be zero in our calculations. This would not be a problem since values as low as this would not be included in our further analysis anyway due to the sampling limitations discussed in GP map analyses for RNA. Once we have a list of low-energy structures for a given sequence, we simply compute the Boltzmann frequencies using Eq. (1) (we did not use ViennaRNA's inbuilt function because we found this to be inaccurate when the isolated basepair setting was switched off when testing our method on short sequences of $L = 15$, where exact calculations with no approximations are feasible).

GP map analyses for RNA

In the last sections, we have described how to fold an individual RNA sequence and obtain its mfe structure and Boltzmann ensemble. However, the full GP map for a sequence length of $L = 30$ contains $4^{30} \approx 10^{18}$ sequences. Thus, we have to rely on sampling approaches to estimate properties like φ_{qp}, f_q, and p_{qp}, as described in the following. φ_{qp} and p_{qp} are both defined as averages over the neutral set of a structure p and are approximated by two samples of 2×10^5 sequences per neutral set (one for φ_{qp} estimates and one for p_{qp} estimates). These samples are generated with Weiß and Ahnert's site-scanning method (25) (parameters: 100 site-scanning processes with 10^5 steps and subsequent subsampling of one in 50 sequences), using a version of this method (22) that includes basepair swaps and is therefore more suitable for sampling from neutral sets, not just their connected components. In the supporting material (section S1.1.1), we provide further details and show that the sample size is large enough to reliably compute φ_{qp} and p_{qp} values $\gtrapprox 10^{-4}$; in the supporting material (section S1.1.2), we further test for systematic bias in the sampling method. Using this sampling method, we computed φ_{qp} and p_{qp} for a total of 50 initial structures p. These were randomly drawn out of all structures obtained from folding 10^8 sequences—the random draw is weighted such that there are a similar number of structures with x stacks. Since the number of stacks is strongly correlated with neutral set sizes (5), which in turn are correlated with robustness (9) and stability (22), this ensures that the chosen structures are qualitatively different. To estimate phenotypic frequencies f_q for Fig. 3, we simply fold 10^8 random sequences and approximate the phenotypic frequency of each structure by its frequency in the sample. This is an unbiased estimator of f_q but inaccurate for $f_q \lessapprox 10^{-8}$, and thus we only plot values of $f_q \geq 5/10^{-8}$. Similarly, we estimated the Boltzmann averages p_q in Fig. 4 by folding 10^7 random sequences (a lower sample size because we get more nuanced quantitative information from each sequence: a Boltzmann ensemble and not just a single structure). Since our 50 selected structures include low-frequency structures (they were selected to represent a range of neutral set sizes), where the requirement $f_q \geq 5/10^{-8}$ does not apply, we estimate their phenotypic frequencies for Fig. 4 with the program by Jörg et al. (24) (with isolated basepairs switched off), which uses a nested Markov chain Monte Carlo algorithm in combination with ViennaRNA predictions. This method does not test for the uniqueness of mfe structures, but the impact of this is minimal for f_q predictions (22). The predictions for structures with $f_q \geq 5/10^{-8}$, where both sampling methods can be used to estimate f_q values, are in excellent agreement between the two methods (see Fig. S5).

HP protein model

Our data rely on a full enumeration of all HP sequences of length $L = 25$ and their folded structures on a compact lattice, using a simple energy model (28) with a stabilising contact energy of one unit for two hydrophobic residues and no energy contribution otherwise. We follow the steps outlined by Greenbury et al. (9,23,29) in the construction of the GP map (including the convention that sequences with multiple mfe structures are considered nonfolding) and test our methods against their data (23). However, in this article, we treat two structures as distinct if they have reversed directionality, i.e., if the structure looks identical except with the N-terminus and C-terminus swapped, as in (30). This convention was chosen for consistency with the RNA folding model, where information on directionality in the folded structure is also retained. The only free parameter in the HP model is the reduced temperature (relative to the HP interaction strength), for which we use $k_BT = 0.5$ since this represents a realistic middle ground between $k_BT = 0.1$ on the one extreme, where the protein has no plasticity and typically spends $>99\%$ of time in the ground state, and $k_BT = 1$ on the other extreme, where the ground state accounts for less than 5% of the Boltzmann ensemble of a typical sequence. However, our results also hold for $k_BT = 0.1$ and 1 (shown in section S7 of the supporting material).

GP map analyses for the HP protein model

f_q and φ_{qp} values are computed exactly. p_{qp} values are approximated by the average over 10^3 sequences drawn with replacement from the neutral set of p. We show in the supporting material (section S1.2) that this sample is large enough for sampling errors to be negligible. In a similar way, Boltzmann averages over arbitrary sequences (p_q) are approximated by the average over 10^5 sequences.

RESULTS

φ_{qp} and Boltzmann frequencies

First, we test both hypotheses in Fig. 2 for one specific RNA structure p (shown in Fig. 3, the structure was chosen to have a median neutral set size: it is the 26th-highest neutral set size out of the 50 structures in our dataset): we plot both the phenotypic frequency f_q (*blue*, Fig. 3 *A*) and the Boltzmann-ensemble-based biophysical quantity p_{qp} (*red*, Fig. 3 *B*) against the corresponding φ_{qp} values. We find that the correlation is much clearer for the biophysical quantity. In order to test if our results generalize beyond the specific RNA structure used in Fig. 3, *A* and *B*, we collected the same data for 50 initial structures p (full data shown in Figs. S9–S13). These 50 structures are very diverse, with between one and five stacks (see GP map analyses for RNA

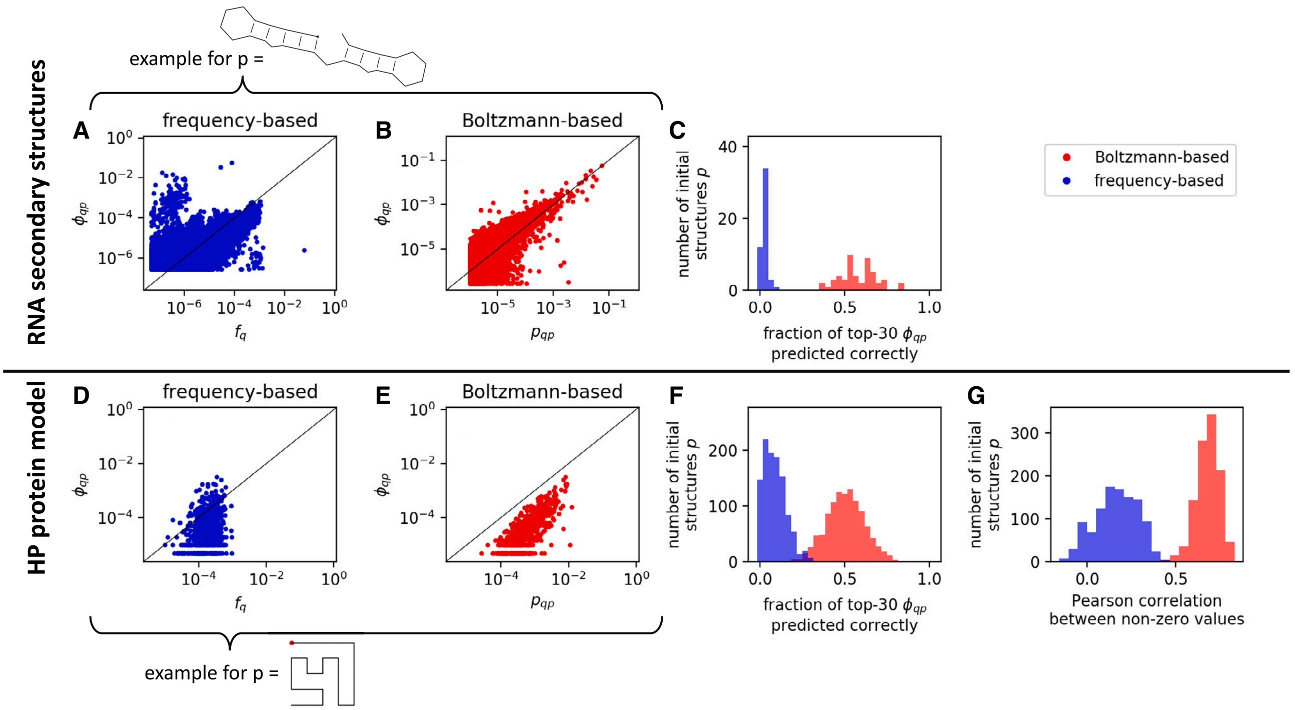

FIGURE 3 Data for the two hypotheses for the RNA secondary structure GP map (*A*–*C*) and the HP protein structure GP map (*D*–*G*). (*A*) Phenotypic-frequency-based hypothesis for one initial RNA structure p as an example (*sketched at the top*): the φ_{qp} values for mutating from this initial structure p to a new structure q are plotted against the phenotypic frequency f_q of the new structure q. (*B*) Biophysical Boltzmann-ensemble-based hypothesis for the same initial RNA structure p: the φ_{qp} values for this initial structure are plotted against the biophysical quantity p_{qp}, i.e., the Boltzmann frequency of the new structure q averaged over sequences with mfe structure p. The black lines indicate $x = y$. (*C*) The analysis is repeated for 50 different initial structures p. For each p, we score each hypothesis by evaluating how many of the structures with the 30 highest φ_{qp} values are captured correctly by each hypothesis. (*D*–*G*) Same analysis for the HP protein model. (*D*) Phenotypic-frequency-based hypothesis for one specific initial structure (*sketched on the bottom*, the *red dot* indicates the start position). (*E*) Biophysical Boltzmann-ensemble-based hypothesis for the same structure. (*F* and *G*) The analysis is repeated for all 1081 HP structures that fold as mfe structures: the biophysical hypothesis scores better, regardless of whether we focus on the top-φ_{qp} structures (*F*) or compute the Pearson correlation coefficients on a log-log scale (*G*). To see this figure in color, go online.

and Fig. S6). Since the number of stacks is correlated with neutral set size (5) and thus with other mutational and biophysical quantities (9, 22), this means that we also have a range of mutational robustness and thermodynamic stability values in our dataset. For each initial structure p, we evaluate how many of the structures with the 30 highest φ_{qp} values are predicted correctly by each of the two hypotheses, based on phenotypic frequencies f_q or the biophysical quantity p_{qp}. We chose a performance metric based on the highest φ_{qp} values for the following reasons: The highest φ_{qp} values are of practical importance since they represent the likeliest mutational transitions, and they are insensitive to sampling errors in our calculations: sampling errors become important around $\varphi_{qp} \lessapprox 10^{-4}$ (see section S1.1.1), whereas the highest φ_{qp} values are typically $\varphi_{qp} \gtrapprox 10^{-2}$, two orders of magnitude higher, and therefore we can determine the highest values with high certainty. Fig. 3 *C* shows this performance metric for our 50 RNA datasets, each corresponding to one initial structure p: we find that, indeed, the biophysical Boltzmann-frequency-based quantity is a better predictor of φ_{qp} than phenotypic frequencies. To understand why phenotypic frequencies f_q might not be a good predictor, we consider the outliers in Fig. 3 *A* in more detail: some of the most likely mutational changes (i.e., high φ_{qp} values) actually correspond to low-f_q structures. These transitions, which are locally frequent from p (i.e., have a high φ_{qp}) but globally rare (i.e., have low f_q), are transitions between two similar structures p and q (see section S2 and (9,15)). These transitions are likely to play a key role in evolutionary processes since incremental variation is more likely to be adaptive than larger structural changes (see (23) for an example). The biophysical Boltzmann-frequency-based approach in Fig. 3 *B* correctly captures these high-φ_{qp} transitions between similar structures. Next, we repeat our analysis for the HP protein model (Fig. 3, *D*–*G*): since the sequence space is smaller in this model, we use exact data without sampling, except for the biophysical quantity p_{qp}. This also means that we have data for all possible initial structures p and for all possible structural changes for each p (see Figs. S14–S18 for further choices of p), and we use all these data in Fig. 3, *F* and *G*. Since sampling errors are not an issue in our HP data, we

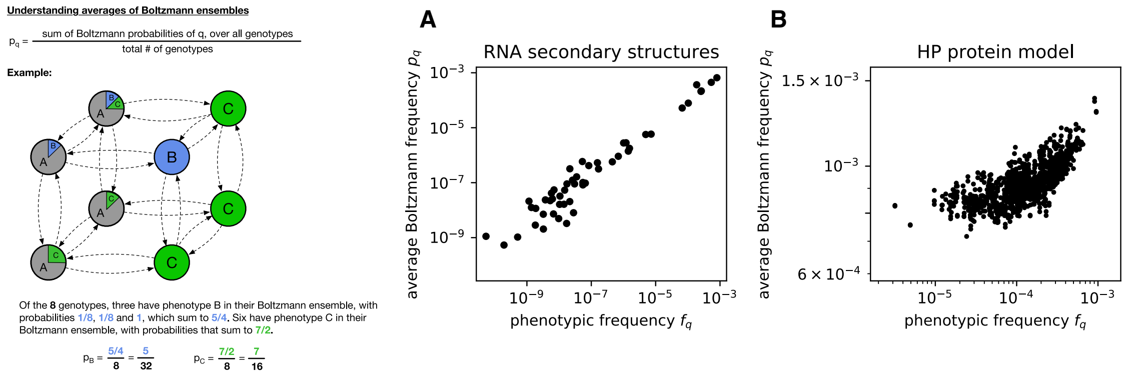

FIGURE 4 Boltzmann frequencies and phenotypic frequencies. Here, we compare phenotypic frequencies f_q to p_q, the average Boltzmann frequency of structure q over all sequences. The definition of p_q is illustrated in the schematic on the left. (*A*) The average Boltzmann frequency p_q of an RNA secondary structure q is plotted against the phenotypic frequency f_q of this structure, i.e., the probability that a random sequence folds into q as its minimum-free-energy structure. (*B*) Same for the HP protein model. The same structures are shown as in Fig. 3, *C* and *F*; the sets of structures and the sampling methods used to estimate f_q and p_q are detailed in the materials and methods. To see this figure in color, go online.

can use the full range of φ_{qp} values and compare the different hypotheses not only based on the highest-φ_{qp} structures (Fig. 3 *F*) but also by computing Pearson correlation coefficients (Fig. 3 *G*). We find that, as before for RNA, the biophysical Boltzmann-frequency-based approach is a better indicator of which structural changes are most likely to occur through mutations. To conclude, neutral set averages of Boltzmann frequencies p_{qp} are a good indicator of which structural transitions are likely to occur through mutations. Here, we have shown that they are a better indicator than phenotypic frequencies since this is the most well-known approximation for φ_{qp}, but in the supporting material (section S4), we also show that they outperform other ways in which φ_{qp} might be estimated, for example based on structural similarity or by using the upper bound given by the conditional complexity arguments from Dingle et al. (15).

Boltzmann frequencies and phenotypic frequencies

To better understand the neutral set averages of Boltzmann frequencies p_{qp}, which are at the center of our analysis, we also evaluated a closely related quantity, namely the averages of Boltzmann frequencies over random sequences. The data in Fig. 4 indicate that this quantity p_q, the mean Boltzmann frequency of structure q over all sequences in the GP map, is correlated with the phenotypic frequency of that structure, especially in the RNA map. This holds regardless of whether mfe structures are included in the averages, as in Fig. 4, or not, as shown in Fig. S28. This result agrees with existing data for RNA (4,31). It is also consistent with theoretical arguments (32) for a Boltzmann-like trend in phenotypic frequencies, where energetically unfavorable features are associated with an exponential decrease in phenotypic frequencies. Not all assumptions behind this theoretical claim are met in our case (for example, that the mean free energy of folding for a random sequence differs from structure to structure, which is not true in many compact HP models (33), and the assumption of constant sequence composition in the random energy model (34)). The fact that not even the key assumption on free-energy distributions holds for the compact HP model may partially explain why the correlation is less clear for the HP model. We can apply the insight from Fig. 4 to neutral set averages as follows: the sequences in a large neutral set are (almost) as diverse as arbitrary sequences, especially for RNA (section S6). Therefore, it is likely that the neutral set averages of Boltzmann frequencies p_{qp} in large neutral sets are similar to the averages over arbitrary sequences p_q. This would mean that for structures p with large neutral sets, we have $p_{qp} \propto f_q$. Thus, we recover the existing hypothesis (9) that for the special case of an initial structure with a large neutral set, there is a high correlation between φ_{qp} and the phenotypic frequency of the new structure f_q.

DISCUSSION

In this article, we have shown that averages over Boltzmann ensembles capture two key GP map quantities: first, if sequences folding into an initial structure p have a structure q as a high-Boltzmann-frequency alternative, then there are many mutations that transform p into q, as previously shown in our data from the RNAshapes model (20). This argument is a better indicator of likely mutational transitions than simply the phenotypic frequency f_q of structure q. Our results thus show that the existing principle of plastogenetic congruence (17) can be generalized from individual sequences to averages over the neutral set of structures, even though the mutational neighborhood (9,12) and the ensemble of suboptimal structures with high Boltzmann frequencies (19) differs markedly from sequence to sequence in a neutral set. Secondly, we found that structures that have high Boltzmann probabilities in arbitrary sequences

also have high phenotypic frequencies f_q, in agreement with previous data for RNA (4,31). Intuitively, plastogenetic congruence should apply for well-behaved energy functions, as in the theoretical calculations in (35), where free energies vary by a small amount after a single mutation. Under these conditions, the mfe structure after a mutation already has to exist as a low-energy structure before the mutation, and so we have plastogenetic congruence. The HP model has such a well-behaved energy function because each residue has up to three contacts, and so the free energy of a given structure can only change by up to three units after a mutation. For RNA structures, however, some mutations will cause free-energy jumps in some structures by preventing a basepair from forming and thus making it impossible for the structure to fold at all. Despite this potential caveat, existing work on plastogenetic congruence (17) in RNA, as well as the data in this article, demonstrates that the concept still holds for RNA. In fact, one classic paper (17) uses basepairing constraints to argue why rare mutational transitions might also be rare plastic transitions. Our results show that the concept holds for both the HP model and the RNA model, indicating that the free-energy jumps caused by basepairing constraints neither prevent nor enable plastogenetic congruence and its generalization to neutral sets. The fact that plastogenetic congruence and similar effects are found in a range of contexts (for example, (36–41)) indicates that our results may also hold more broadly for a range of GP maps. However, many of these analyses focus on continuous phenotypic and structural changes, whereas here, we have focused specifically on the probabilities of obtaining specific discrete structural changes because these probabilities have been shown to be highly biased (11) and impact evolutionary processes (1). Future work should investigate connections between these different perspectives on mutational changes. One implication of our results is that structures that have high average Boltzmann probabilities and are thus likely to evolve as suboptimal structures also have high φ_{qp} values and are thus more likely to evolve as mfe structures, as conjectured in Ancel and Fontana's original paper on plastogenetic congruence (17). This applies for arbitrary sequences, where the relevant quantities are f_q and p_q, and for a given initial structure, where the relevant quantities are φ_{qp} and p_{qp}. This finding is highly relevant in cases where the fitness of a sequence is not determined simply by the identity of its mfe structure but instead depends on the Boltzmann frequency of the correctly folded structure, as was found in a large-scale experimental study on tRNAs (42). In this case, the likelihood of evolving a given structure q would not be given by φ_{qp} and f_q values, as traditionally assumed in models (1,5,11,15,43), but by a quantity that reflects the possibility that q could emerge as a suboptimal structure, i.e., a quantity related to the Boltzmann averages p_q or p_{qp}. Given the progress in calculating the probabilities and timescales on which different structures evolve in the simpler models where only mfe structures matter (1,2,3), future work should investigate these questions systematically for cases in which the fitness depends on suboptimal structures. In this case, the established definition of φ_{qp} should be adjusted to include these suboptimal structures of each sequence, similar to the rates in (44). Our data on the parallels between Boltzmann ensembles and mutational changes can guide such analyses. Furthermore, our results could have a practical application in sampling methods: usually, GP map analyses are restricted to short sequences because of the computational cost required to sample a sufficient number of sequences to estimate quantities like phenotypic frequencies and mutation probabilities φ_{qp}. Sophisticated methods that reduce the required sample sizes are therefore important (24,25). Relying on the biophysical quantity p_{qp} could be one option of reducing sampling sizes when estimating φ_{qp} data: if φ_{qp} was estimated directly from a sequence sample, one needs to fold all the sequences in the sample and their mutational neighbors, but for the biophysical quantity p_{qp}, one only needs to obtain the Boltzmann ensemble of the sequences itself. This is especially useful in cases like the HP model, where entire Boltzmann ensembles can be predicted as quickly as the mfe structure since the mfe structure is usually identified by computing the energy of all folds (for example, (8,29)). It may even be possible to develop methods that estimate p_{qp} without extensive sampling by building on existing techniques (45,46) developed for bistable sequences, i.e., sequences that fold into both p and q with high probabilities. Potential avenues for future research in this direction are discussed in more detail in section S8 of the supporting material. In a similar way, the results may be useful when making inferences from partial experimental data on mutational changes and fluctuations.

DATA AND CODE AVAILABILITY

The code behind this analysis can be found at https://github.com/noramartin/mutation_probabilities.

SUPPORTING MATERIAL

Supporting material can be found online at https://doi.org/10.1016/j.bpj.2023.10.024.

AUTHOR CONTRIBUTIONS

N.S.M. designed and performed the computational analysis and wrote the manuscript. S.E.A. supervised the project.

ACKNOWLEDGMENTS

The authors would like to thank J. Blundell and T. McLeish for discussions. N.S.M. acknowledges funding from the Gates Cambridge Trust and the Winton Programme for the Physics of Sustainability and the Issachar Fund. S.E.A. was supported by the Gatsby Charitable Foundation with grant no. PTAG/021.

DECLARATION OF INTERESTS

The authors declare no competing interests.

SUPPORTING CITATIONS

References (47–59) appear in the Supporting Material.

REFERENCES

1. Schaper, S., and A. A. Louis. 2014. The arrival of the frequent: how bias in genotype-phenotype maps can steer populations to local optima. *PLoS One.* 9, e86635.
2. Cowperthwaite, M. C., E. P. Economo, …, L. A. Meyers. 2008. The ascent of the abundant: how mutational networks constrain evolution. *PLoS Comput. Biol.* 4, e1000110.
3. Stich, M., and S. C. Manrubia. 2011. Motif frequency and evolutionary search times in RNA populations. *J. Theor. Biol.* 280:117–126.
4. Dingle, K., F. Ghaddar, …, A. A. Louis. 2022. Phenotype bias determines how natural RNA structures occupy the morphospace of all possible shapes. *Mol. Biol. Evol.* 39:msab280.
5. Dingle, K., S. Schaper, and A. A. Louis. 2015. The structure of the genotype–phenotype map strongly constrains the evolution of non-coding RNA. *Interface focus.* 5, 20150053.
6. Manrubia, S., J. A. Cuesta, …, M. Weiß. 2021. From genotypes to organisms: State-of-the-art and perspectives of a cornerstone in evolutionary dynamics. *Phys. Life Rev.* 38:55–106.
7. Lorenz, R., S. H. Bernhart, …, I. L. Hofacker. 2011. ViennaRNA Package 2.0. *Algorithm Mol. Biol.* 6:26.
8. Li, H., R. Helling, …, N. Wingreen. 1996. Emergence of preferred structures in a simple model of protein folding. *Science.* 273:666–669.
9. Greenbury, S. F., S. Schaper, …, A. A. Louis. 2016. Genetic correlations greatly increase mutational robustness and can both reduce and enhance evolvability. *PLoS Comput. Biol.* 12, e1004773.
10. Stadler, B. M., P. F. Stadler, …, W. Fontana. 2001. The topology of the possible: Formal spaces underlying patterns of evolutionary change. *J. Theor. Biol.* 213:241–274.
11. Fontana, W., and P. Schuster. 1998. Continuity in evolution: on the nature of transitions. *Science.* 280:1451–1455.
12. Wagner, A. 2008. Robustness and evolvability: a paradox resolved. *Proc. Biol. Sci.* 275:91–100.
13. Manrubia, S., and J. A. Cuesta. 2015. Evolution on neutral networks accelerates the ticking rate of the molecular clock. *J. R. Soc., Interface.* 12, 20141010.
14. Vīksna, J., and D. Gilbert. 2007. Assessment of the probabilities for evolutionary structural changes in protein folds. *Bioinformatics.* 23:832–841.
15. Dingle, K., J. K. Novev, …, A. A. Louis. 2022. Predicting phenotype transition probabilities via conditional algorithmic probability approximations. *J. R. Soc., Interface.* 19, 20220694.
16. Wuchty, S., W. Fontana, …, P. Schuster. 1999. Complete suboptimal folding of RNA and the stability of secondary structures. *Biopolymers.* 49:145–165.
17. Ancel, L. W., and W. Fontana. 2000. Plasticity, evolvability, and modularity in RNA. *J. Exp. Zool.* 288:242–283.
18. Derényi, I., and G. J. Szöllősi. 2015. Effective Temperature of Mutations. *Phys. Rev. Lett.* 114, 058101.
19. Wagner, A. 2014. Mutational robustness accelerates the origin of novel RNA phenotypes through phenotypic plasticity. *Biophys. J.* 106:955–965.
20. Martin, N. S., and S. E. Ahnert. 2021. Insertions and deletions in the RNA sequence–structure map. *J. R. Soc., Interface.* 18, 20210380.
21. Turner, D. H., and D. H. Mathews. 2010. NNDB: the nearest neighbor parameter database for predicting stability of nucleic acid secondary structure. *Nucleic Acids Res.* 38:D280–D282.
22. Martin, N. S., and S. E. Ahnert. 2022. Thermodynamics and neutral sets in the RNA sequence-structure map. *EPL.* 139, 37001.
23. Greenbury, S. F., A. A. Louis, and S. E. Ahnert. 2022. The structure of genotype-phenotype maps makes fitness landscapes navigable. *Nat. Ecol. Evol.* 6:1742–1752.
24. Jörg, T., O. C. Martin, and A. Wagner. 2008. Neutral network sizes of biological RNA molecules can be computed and are not atypically small. *BMC Bioinf.* 9:464.
25. Weiß, M., and S. E. Ahnert. 2020. Using small samples to estimate neutral component size and robustness in the genotype–phenotype map of RNA secondary structure. *J. R. Soc., Interface.* 17, 20190784.
26. Rezazadegan, R., C. Barrett, and C. Reidys. 2018. Multiplicity of phenotypes and RNA evolution. *J. Theor. Biol.* 447:139–146.
27. Nebel, M. E., and A. Scheid. 2009. On quantitative effects of RNA shape abstraction. *Theor. Biosci.* 128:211–225.
28. Irbäck, A., and C. Troein. 2002. Enumerating designing sequences in the HP model. *J. Biol. Phys.* 28:1–15.
29. Greenbury, S. F. 2014. General Properties of Genotype-Phenotype Maps for Biological Self-Assembly. Ph.D. thesis, University of Cambridge.
30. Bornberg-Bauer, E. 1997. How are model protein structures distributed in sequence space? *Biophys. J.* 73:2393–2403.
31. García-Galindo, P., S. E. Ahnert, and N. S. Martin. 2023. The non-deterministic genotype–phenotype map of RNA secondary structure. *J. R. Soc., Interface.* 20, 20230132.
32. Finkelstein, A. V., A. Y. Badretdinov, and A. M. Gutin. 1995. Why do protein architectures have Boltzmann-like statistics? *Proteins.* 23:142–150.
33. Li, H., C. Tang, and N. S. Wingreen. 1998. Are protein folds atypical? *Proc. Natl. Acad. Sci. USA.* 95:4987–4990.
34. Shakhnovich, E. I., and A. M. Gutin. 1993. Engineering of stable and fast-folding sequences of model proteins. *Proc. Natl. Acad. Sci. USA.* 90:7195–7199.
35. Tang, Q.-Y., and K. Kaneko. 2021. Dynamics-evolution correspondence in protein structures. *Phys. Rev. Lett.* 127, 098103.
36. Sato, K., Y. Ito, …, K. Kaneko. 2003. On the relation between fluctuation and response in biological systems. *Proc. Natl. Acad. Sci. USA.* 100:14086–14090.
37. Furusawa, C., and K. Kaneko. 2015. Global relationships in fluctuation and response in adaptive evolution. *J. R. Soc., Interface.* 12, 20150482.
38. Espinosa-Soto, C., O. C. Martin, and A. Wagner. 2011. Phenotypic plasticity can facilitate adaptive evolution in gene regulatory circuits. *BMC Evol. Biol.* 11:5.
39. Tawfik, D. S. 2010. Messy biology and the origins of evolutionary innovations. *Nat. Chem. Biol.* 6:692–696.
40. Leo-Macias, A., P. Lopez-Romero, …, A. R. Ortiz. 2005. An analysis of core deformations in protein superfamilies. *Biophys. J.* 88:1291–1299.
41. Brun-Usan, M., A. Rago, …, R. A. Watson. 2021. Development and selective grain make plasticity 'take the lead' in adaptive evolution. *BMC Ecol. Evol.* 21:205.
42. Li, C., W. Qian, …, J. Zhang. 2016. The fitness landscape of a tRNA gene. *Science.* 352:837–840.
43. Cowperthwaite, M. C., and L. A. Meyers. 2007. How mutational networks shape evolution: Lessons from RNA models. *Annu. Rev. Ecol. Evol. Syst.* 38:203–230.
44. Coluzza, I., J. T. MacDonald, …, R. A. Goldstein. 2012. Analytic markovian rates for generalized protein structure evolution. *PLoS One.* 7, e34228.
45. Taneda, A. 2015. Multi-objective optimization for RNA design with multiple target secondary structures. *BMC Bioinf.* 16:280.

46. Hammer, S., W. Wang, …, Y. Ponty. 2019. Fixed-parameter tractable sampling for RNA design with multiple target structures. *BMC Bioinf.* 20:209.

47. Hofacker, I. L. 2009. RNA secondary structure analysis using the Vienna RNA Package. *Curr. Protoc. Bioinf.* 12:12.2.1–12.2.16.

48. Buchler, N. E. G., and R. A. Goldstein. 2000. Surveying determinants of protein structure designability across different energy models and amino-acid alphabets: A consensus. *J. Chem. Phys.* 112:2533–2547.

49. Wroe, R., H. S. Chan, and E. Bornberg-Bauer. 2007. A structural model of latent evolutionary potentials underlying neutral networks in proteins. *HFSP J.* 1:79–87.

50. Steger, G., and R. Giegerich. 2013. 14. RNA structure prediction. *In* RNA Structure and Folding. D. Klostermeier and C. Hammann, eds De Gruyter, Berlin, Boston, pp. 335–362.

51. Godzik, A., J. Skolnick, and A. Kolinski. 1993. Regularities in interaction patterns of globular proteins. *Protein Eng.* 6:801–810.

52. Ferrada, E., and A. Wagner. 2012. A comparison of genotype-phenotype maps for RNA and proteins. *Biophys. J.* 102:1916–1925.

53. Bastolla, U., H. E. Roman, and M. Vendruscolo. 1999. Neutral evolution of model proteins: diffusion in sequence space and overdispersion. *J. Theor. Biol.* 200:49–64.

54. Schuster, P., W. Fontana, …, I. L. Hofacker. 1994. From sequences to shapes and back: a case study in RNA secondary structures. *Proc. R. Soc. A B.* 255:279.

55. Mathews, D. H., and D. H. Turner. 2006. Prediction of RNA secondary structure by free energy minimization. *Curr. Opin. Struct. Biol.* 16:270–278.

56. Bloom, J. D., J. J. Silberg, …, F. H. Arnold. 2005. Thermodynamic prediction of protein neutrality. *Proc. Natl. Acad. Sci. USA.* 102:606–611.

57. Porter, L. L., and L. L. Looger. 2018. Extant fold-switching proteins are widespread. *Proc. Natl. Acad. Sci. USA.* 115:5968–5973.

58. García-Martín, J. A., P. Catalán, …, J. A. Cuesta. 2018. Statistical theory of phenotype abundance distributions: A test through exact enumeration of genotype spaces. *EPL.* 123, 28001.

59. Jacquin, H., A. Gilson, …, R. Monasson. 2016. Benchmarking inverse statistical approaches for protein structure and design with exactly solvable models. *PLoS Comput. Biol.* 12, e1004889.

Biophysical Journal, Volume *122*

Supplemental information

The Boltzmann distributions of molecular structures predict likely changes through random mutations

Nora S. Martin and Sebastian E. Ahnert

Supplementary Information: The Boltzmann distributions of folded molecular structures predict likely changes through random mutations

Nora S. Martin, Sebastian E. Ahnert

Contents

S1 Test of sampling methods **2**
S1.1 Test of sampling methods: RNA 2
S1.1.1 Test for sample size with sub-sampling 2
S1.1.2 Test for systematic bias 5
S1.1.3 Comparison of two different sampling methods for phenotypic frequencies 6
S1.1.4 Structural diversity in the sample of initial structures 7
S1.2 Test of sampling methods: HP model 7

S2 Mutation probabilities, phenotypic frequencies and structural similarities **9**

S3 Main hypothesis comparison - data for further initial structures **9**
S3.1 RNA secondary structures 9
S3.2 HP protein model 15

S4 Alternative approach: structural similarity as an indicator of mutation probabilities **20**
S4.1 RNA secondary structures 20
S4.1.1 RNA structural distance quantified by the base pair distance 20
S4.1.2 RNA structural distance quantified in terms of shared compatible sequences 20
S4.1.3 RNA structural distance quantified using conditional complexities 21
S4.1.4 Systematic comparison of potential ϕ_{qp} indicators 22
S4.2 HP protein model 26
S4.2.1 HP structural distance quantified by the contact map overlap 26
S4.2.2 HP structural distance quantified by the difference in core/surface profiles 27
S4.2.3 HP structural distance quantified using conditional complexities 28
S4.2.4 Systematic comparison of potential ϕ_{qp} indicators 30

S5 Average Boltzmann frequencies p_q and phenotypic frequencies f_q - without mfe structures **31**

S6 Sequence diversity in neutral sets **32**

S7 Different reduced temperature parameters in the HP model **35**

S8 Outline of potential applications in sampling techniques **36**

S1 Test of sampling methods

S1.1 Test of sampling methods: RNA

ϕ_{qp} and p_{qp} are defined as averages over the neutral set of a structure p. However, neutral set sizes for RNA structures can contain $\gtrapprox 10^{15}$ sequences[1]. Therefore, our ϕ_{qp} and p_{qp} values are estimated based on a fixed-sized sequence sample from the relevant neutral set: we generate two samples of 2×10^5 sequences for the neutral set of each initial structure p, using an adaptation of Weiß and Ahnert's [1] site-scanning method, as described in the main text. We then use one of these samples to estimate ϕ_{qp} and the second sample to estimate p_{qp}. In this section, we perform two tests of this sampling method: first, we use sub-sampling to investigate whether the sample size is large enough (section S1.1.1). Secondly, we use an alternative sampling method to check for systematic biases (section S1.1.2).

S1.1.1 Test for sample size with sub-sampling

In order to exclude data points that cannot be estimated reliably with the given sample size, we only include a ϕ_{qp} value in our analysis and plots if it is based on at least 5 instances of q (i.e. in total there are five mutations from any of the sequences in the sample to q), and a p_{qp} value if the numerator of the calculation is at least 0.2 (i.e. $p_{qp} \geq 0.2/(2 \times 10^5)$).
Nevertheless, we checked if the finite sample size affects our ϕ_{qp} and p_{qp} values by using sub-sampling (as in our previous paper [2]). For each neutral set p, we compared the ϕ_{qp} and p_{qp} values obtained from the full sequence sample against those calculated from only 10% of the sequence sample (Figs. S1-S2). ϕ_{qp} and p_{qp} values of $\gtrapprox 10^{-4}$ are not strongly affected by the subsampling, which indicates that our sample size is large enough to infer these values reliably.

[1]This can be seen in Fig. 4 in the main text: if one of $\approx 10^3$ sequences folds into a given structure, and there are 4^{30} sequences of $L = 30$, then there are $\approx 10^{15}$ sequences for that structure.

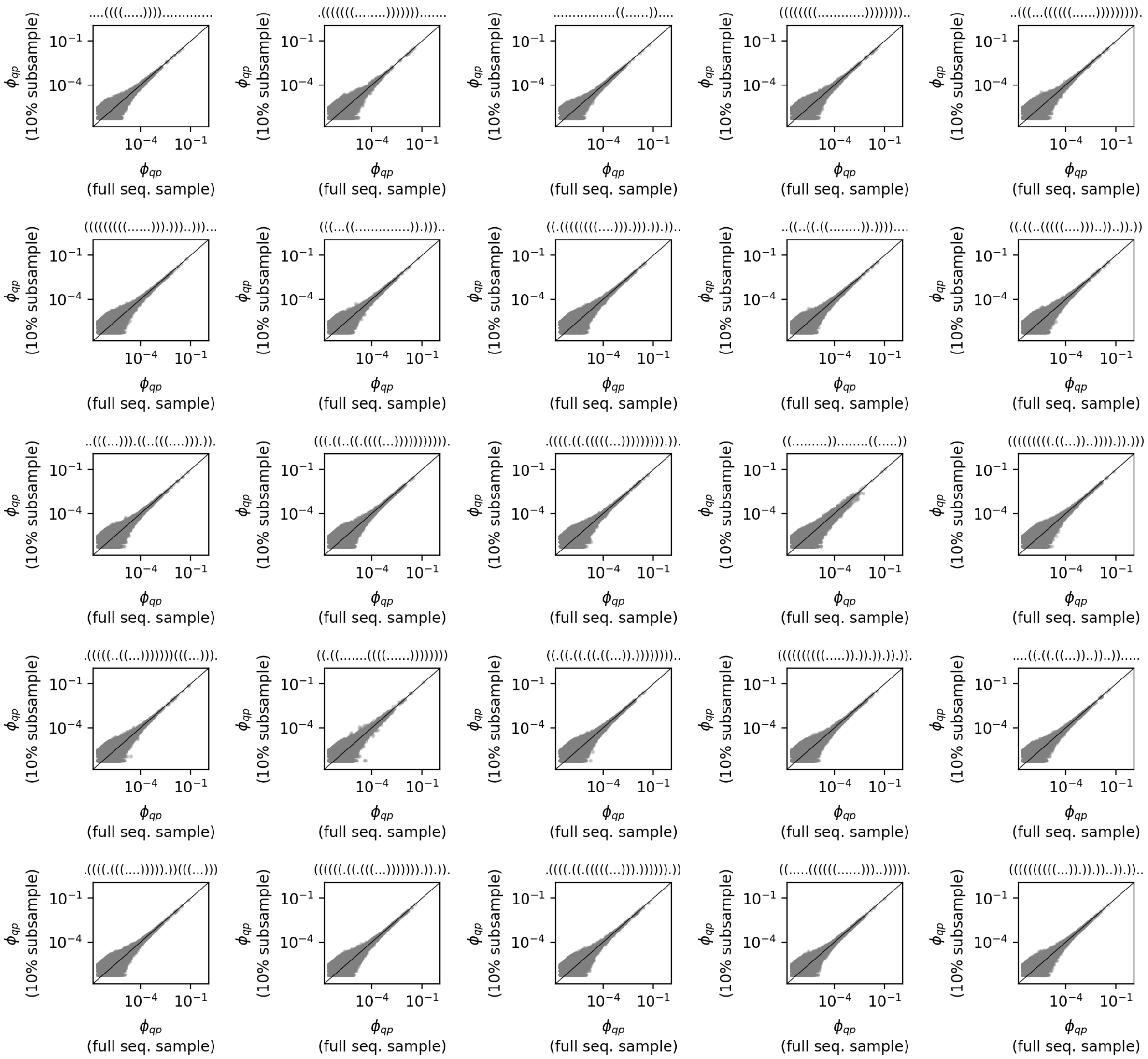

Figure S1: ***Using subsampling to test if our sequence samples are large enough to infer ϕ_{qp} values:*** *each subplot focuses on the neutral set of one initial structure p, which is given in the subplot title in dot-bracket notation (every second structure from a frequency-ordered list of the 50 structures in our dataset, the highest-frequency structures are shown first). In each case, the ϕ_{qp} values inferred from the full sequence sample of 2×10^5 sequences is plotted against those inferred from a random subsample of 2×10^4 sequences (i.e. 10% of the full sample). We find that ϕ_{qp} values $\gtrapprox 10^{-4}$ are robust to subsampling, indicating that the sample size is sufficiently large to infer these values reliably.*

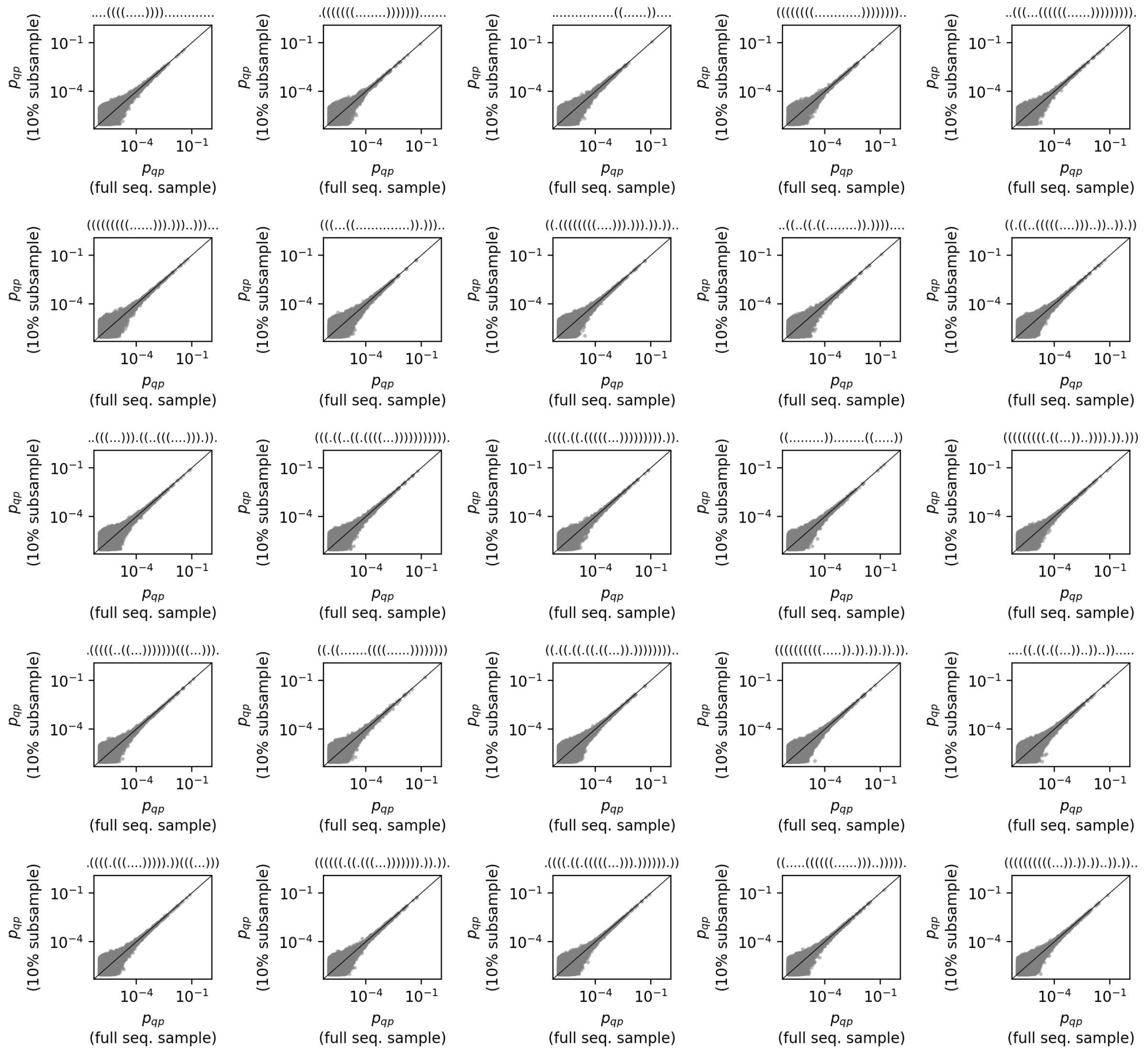

Figure S2: ***Using subsampling to test if our sequence samples are large enough to infer*** p_{qp} ***values:*** *same as Fig. S1 but for* p_{qp} *instead of* ϕ_{qp}*.*

S1.1.2 Test for systematic bias

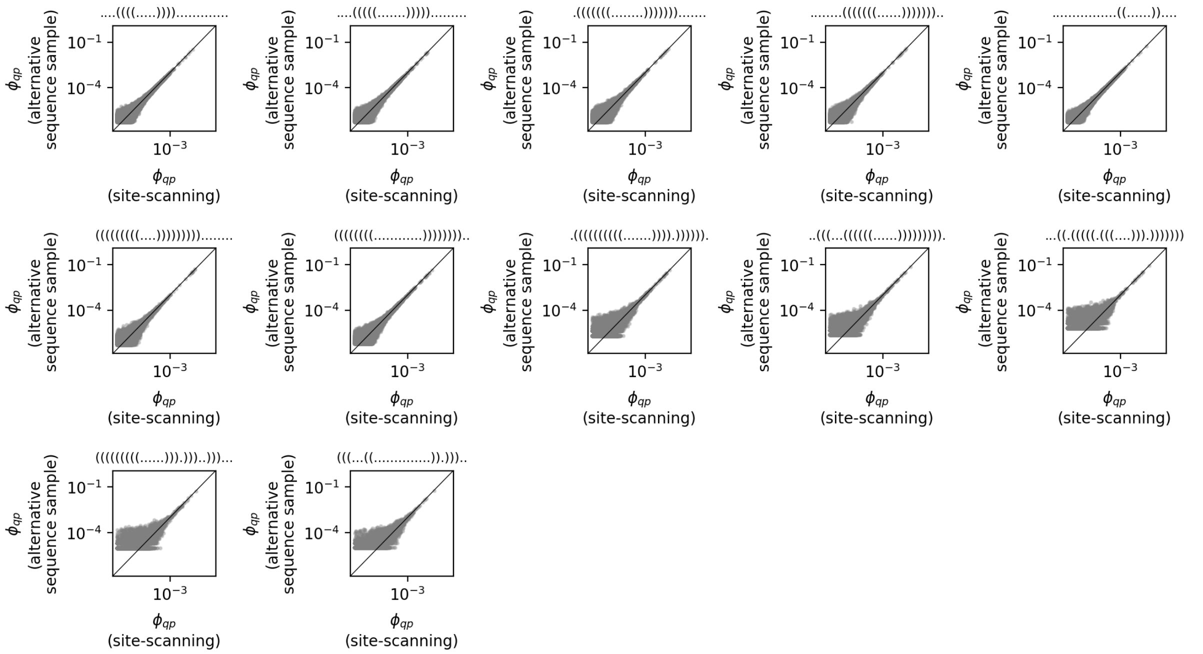

Figure S3: ***Comparison of the ϕ_{qp} data for RNA from two sampling approaches:*** *in order to test our sample-based ϕ_{qp} data, we compute ϕ_{qp} from two different sequence samples based on two different approaches (an adaptation of Weiß and Ahnert's [1] site-scanning method and a simple unbiased brute-force approach, as described in the text). Each subplot shows the ϕ_{qp} data for a given initial structure p, which is given in the subplot title. The plot only includes structures, for which the brute-force approach generated $\geq 10^3$ sequences, and therefore only a subset of the 50 structures p in our sample are shown. The structures are shown in order of decreasing phenotypic frequency, such that the structures for which the alternative approach is expected to work best appear first. We find that the ϕ_{qp} values from the two methods are in good agreement, with the exception of sampling errors, which we expect to affect values of $\lessapprox 10^{-3}$ due to the smaller sample sizes of $\geq 10^3$ sequences in the brute-force method.*

To ensure that our sequence sampling method introduced no systematic biases, we adapt an analysis in the SI of our previous paper [2]: we compare our ϕ_{qp} and p_{qp} values against those derived from a different type of sequence sample. To obtain this second sequence sample for the neutral set of a given p, we simply generate a large number of 10^9 random sequences and only keep those that fall within the neutral set of p. This approach is guaranteed to generate an unbiased sequence sample from the neutral set, but this sample may be too small if the neutral set is small: if we require $\approx 10^4$ sequences in our sample, it will work for initial phenotypes p with $f_p \gtrapprox 10^4/10^9 = 10^{-5}$.

In Figs. S3 & S4, we show the data for all neutral sets, for which the alternative method generated at least 10^3 sequences[2]. This comparison shows that the ϕ_{qp} and p_{qp} values are in good agreement for values $> 10^{-3}$, which can be estimated reliably from the relevant sample sizes. Since we have ordered the subplots by phenotypic frequency in descending order, the first subplots are based on larger samples, are therefore less affected by sample size issues and show a clearer agreement.

[2] If more than 10^5 sequences are obtained for a particular structure p, we only keep the first 10^5 for computational reasons. The additional accuracy obtained from a larger sample would not be useful for our purposes since we are comparing against the site-scanning sample, which has 2×10^5 sequences per structure.

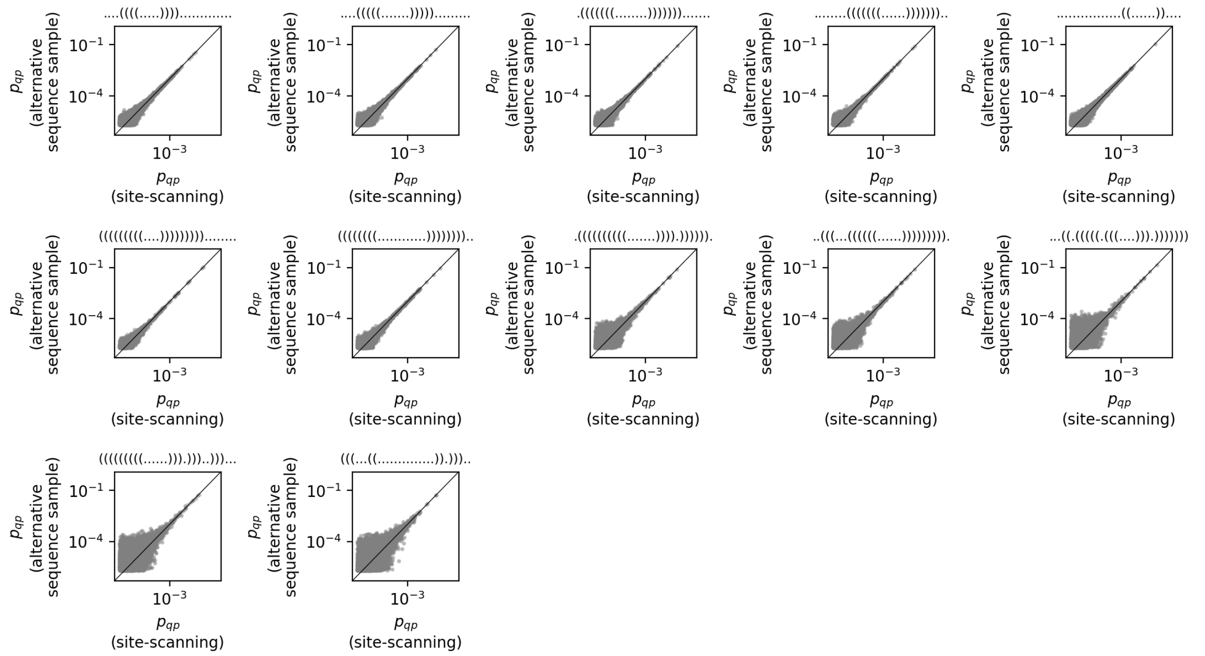

Figure S4: ***Comparison of the p_{qp} data for RNA from two sampling approaches:*** *same as Fig. S3 but for p_{qp} instead of ϕ_{qp}.*

S1.1.3 Comparison of two different sampling methods for phenotypic frequencies

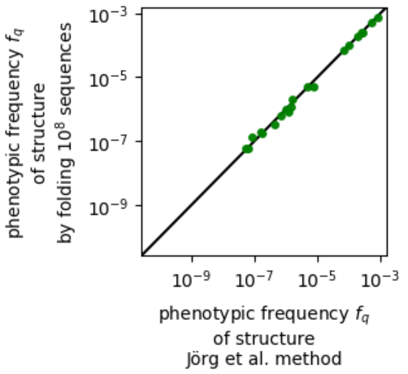

Figure S5: ***Phenotypic frequencies for our set of*** 50 ***initial structures estimated using two different methods:*** *Jörg et al.'s [3] neutral set size estimator (x-axis) and the inferred frequency from folding* 10^8 *random sequences. Since the latter method only works for structures above a certain frequency threshold, only* 20 *out of the* 50 *structures are found. We find excellent agreement between the two methods, even though they are based on different principles.*

There is another case, where we can compare the data from two entirely different sampling methods: the phenotypic frequencies for our 50 selected structures were estimated with Jörg et al.'s [3] neutral set size estimator, which is based on a nested Markov Chain Monte Carlo approach. However, we also have phenotypic frequency estimates for all structures with $f_p \geq 5 \times 10^{-8}$, which we obtained by folding 10^8 sequences and computing the frequency of each structure in the resulting sample. Fig. S5 demonstrates that both methods are in excellent agreement .

S1.1.4 Structural diversity in the sample of initial structures

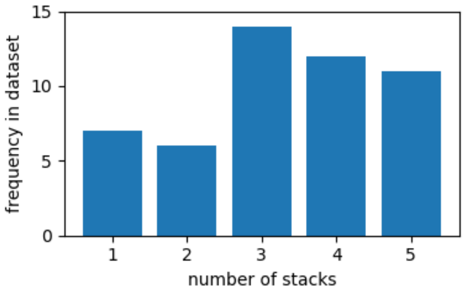

Figure S6: ***Distribution of the number of stacks in the set of*** 50 ***RNA secondary structures used as initial structures*** p ***in our*** ϕ_{qp} ***analyses.***

Finally, we investigate whether the 50 RNA secondary structures used as initial structures p in our ϕ_{qp} analyses are sufficiently diverse. We want them to have a range of values for the number of stacks, since this is known to be linked to their role in the GP map (especially their neutral set sizes [4] and thus their robustness [5] and evolvability [6]) and also linked to their biophysical quantities (especially stability in the Boltzmann ensemble [7]). Thus having structures with a range of stack numbers, as shown in Fig. S6 guarantees that the structures have a range of biophysical properties and a range of roles in the GP map. We have structures with between one and five stacks in our data - note that five stacks is a high number for sequences with $L = 30$ bases because even a short stack takes up a few bases: each stack consists of two base-pairing strands, and there must usually be unpaired regions separating two stacks from one another (such as hairpin loops, internal loops etc).

S1.2 Test of sampling methods: HP model

In our data for the HP protein model, there are fewer sequences in the GP map and so most quantities are computed exactly and sampling methods are only employed for averages over Boltzmann frequencies. Because the structure of every sequence is known, we can enumerate all sequences in a neutral set and randomly draw from this list. Thus, there is no systematic bias in the sequence samples and in the p_{qp} estimates based on these samples, and we only need to test whether the sample size of 10^3 sequences per neutral set is sufficiently large. Again, we compare our values against those computed from a smaller sample of 10^2 sequences to investigate this (Fig S7). We find that p_{qp} values are robust to whether they are computed from the full sequence sample or a smaller sample, indicating that the sample size is sufficiently large to infer these values reliably.

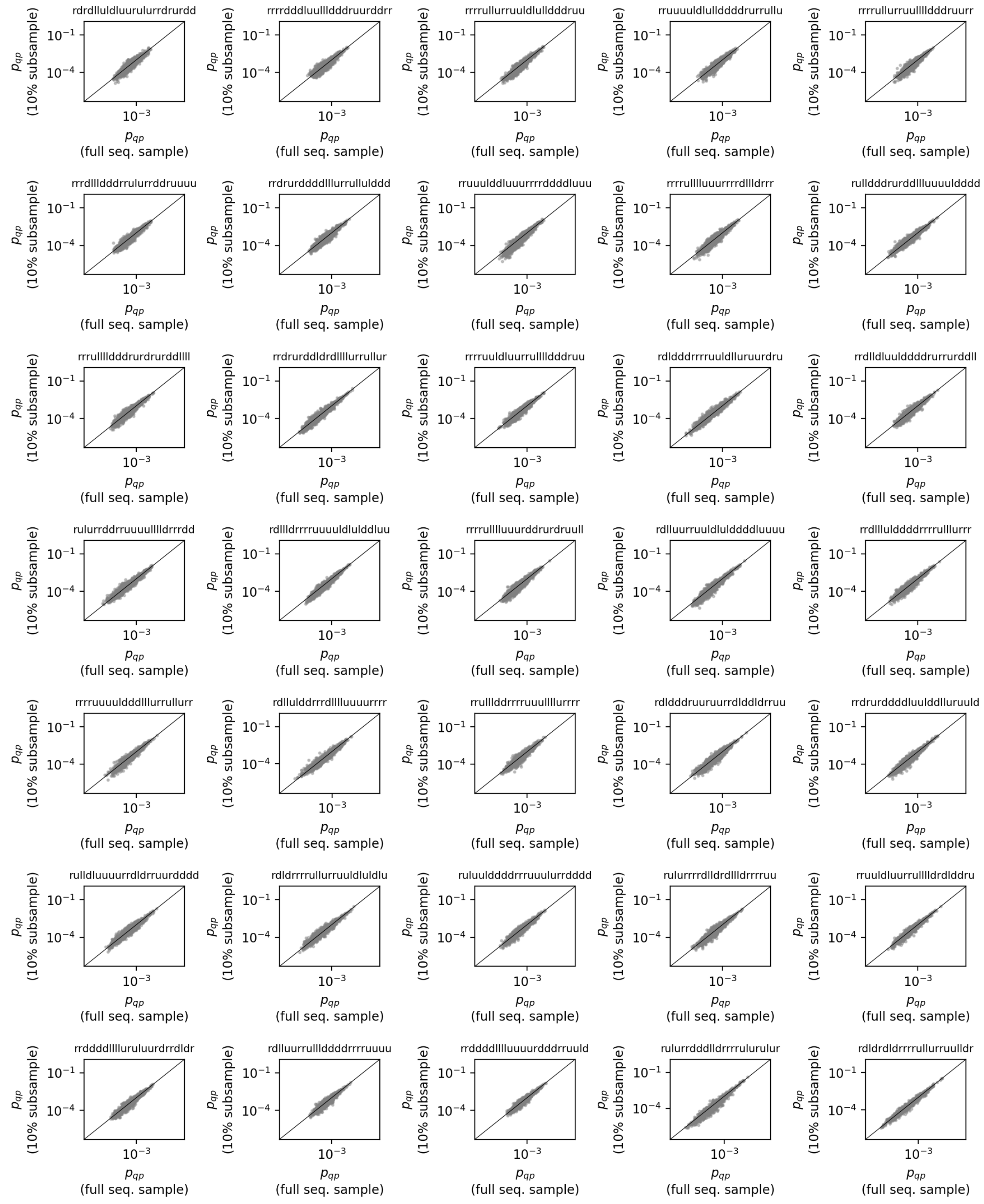

Figure S7: ***Using subsampling to test if our sequence samples are large enough to infer p_{qp} values in the HP model:*** *each subplot focuses on the neutral set of one initial structure p, which is given in the subplot title (the structures are selected in evenly spaced intervals from a full list of HP structures, ranked in descending order by phenotypic frequency, such that both frequent and rare structures appear in the plot). Here we use the following convention for representing 2D lattice structures as strings: we walk along the structure and record "u" for a step upwards on the grid, 'd" for a step down and "r/l" for right or left. For each initial structure p, the p_{qp} values inferred from a random subsample of 10^2 sequences (i.e. 10% of the full sample) are plotted against those inferred from the full sequence sample of 10^3 sequences. We find that p_{qp} values are robust to subsampling, indicating that the sample size is sufficiently large to infer these values reliably.*

S2 Mutation probabilities, phenotypic frequencies and structural similarities

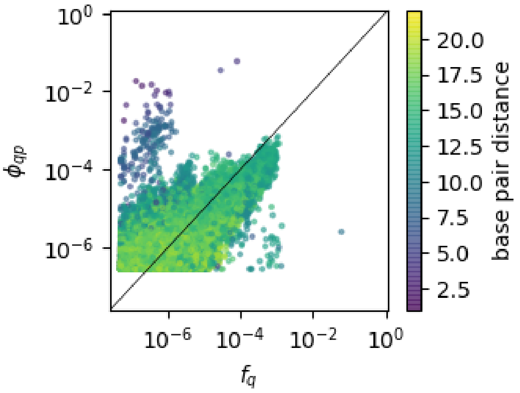

Figure S8: ***Outliers in the phenotypic-frequency-based approach correspond to incremental structural changes:*** *here, the mutation probability ϕ_{qp} is plotted against the phenotypic frequency f_q for one specific initial structure p (same data as Fig 3A in the main text). The colour of each scatter point indicates the number of base pairs, in which the final structure q differs from the initial structure p (using the base pair distance implementation from the ViennaRNA package [8]). We find that the high-ϕ_{qp}-low-f_q outliers correspond to structural transitions between structures that only differ in a small number of base pairs.*

In the main text, we showed data for the phenotypic-frequency-based approach by plotting the mutation probability ϕ_{qp} against the phenotypic frequency f_q. For the RNA model, we observed a positive trend, but with clear outliers: structures with high mutation probabilities ϕ_{qp}, but low phenotypic frequencies f_q. Fig S8 shows that these outliers correspond to structural transitions between structures that only differ in a small number of base pairs, in agreement with ref [9]. This might be expected intuitively [5] and forms the basis for the conditional-complexity approach [10] discussed in the next section. Thus, structures that are similar to p are much easier to evolve from p (i.e. high ϕ_{qp}) than they are to evolve from a random genotype (i.e. low f_q). This could be important for evolutionary processes as it enables structural evolution to proceed incrementally, as illustrated by the examples in ref [11].

S3 Main hypothesis comparison - data for further initial structures

S3.1 RNA secondary structures

In the main text, we compared two hypotheses: first, that the phenotypic frequency f_q is the best indicator of mutation probabilities ϕ_{qp} and secondly that a biophysical quantity, the mean Boltzmann average of q in the neutral set of structure p, is the best indicator of mutation probabilities ϕ_{qp}. In the main text, we had space to compare these two approaches for one choice of initial structure p (Figs. 3 A & B in the main text), and only reported summary statistics for a broader sample of 50 structures with a diverse range of properties. In Figs. S9-S13, we show the full data for these 50 structures using the same format as in the main text: the left column shows the frequency-based approach, the right column the corresponding data for the biophysical Boltzmann-ensemble-based approach. Each row gives the ϕ_{qp} values for one specific initial structure p. These plots confirm the conclusions from the main text: the biophysical approach is a much better indicator of p_{qp} differences.

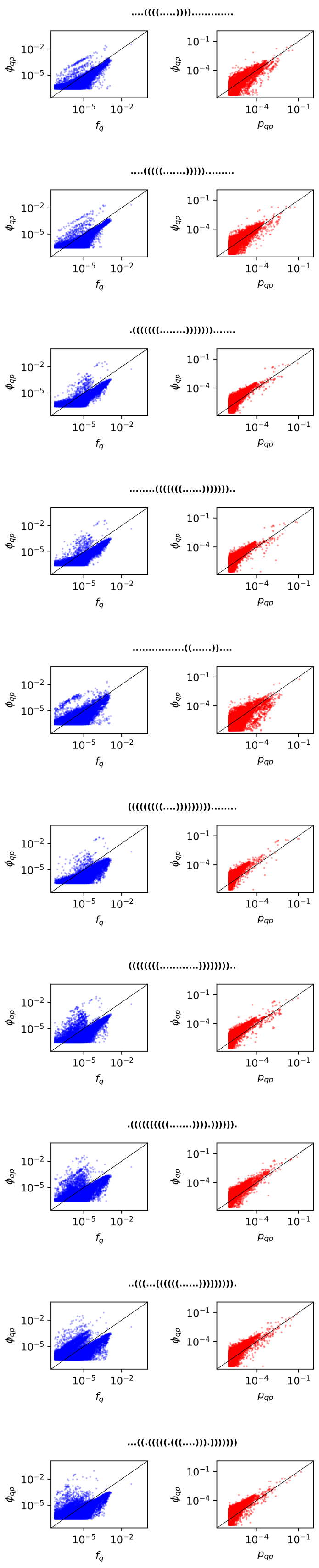

Figure S9: ***Further data for all*** 50 ***initial RNA structures*** p ***in our dataset:*** *each initial structure* p *is shown in a separate row and given in the row title in dot-bracket notation, and the structures are sorted by frequency in descending order. Otherwise, the figure has exactly the same format as Figs. 3A & B in the main text. Continued in Figs S10-S13.*

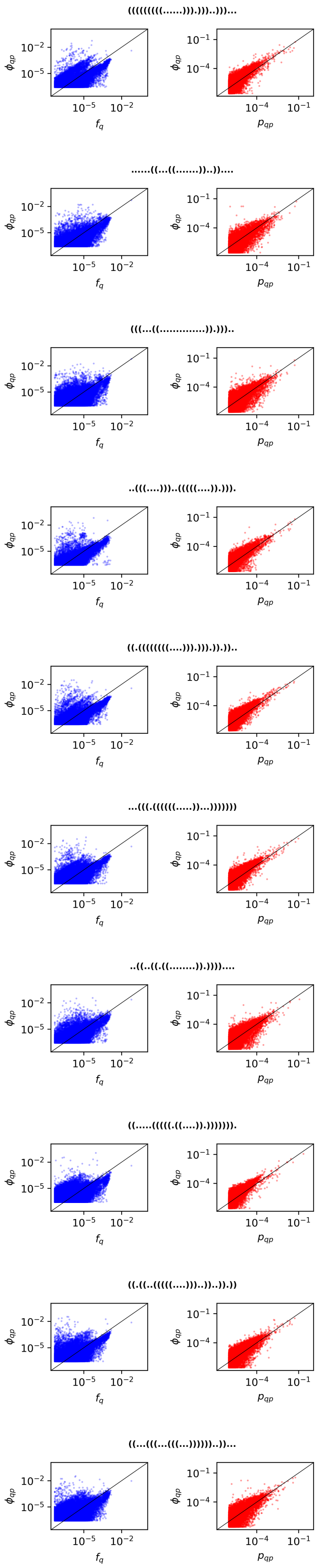

Figure S10: Continued from Fig S9

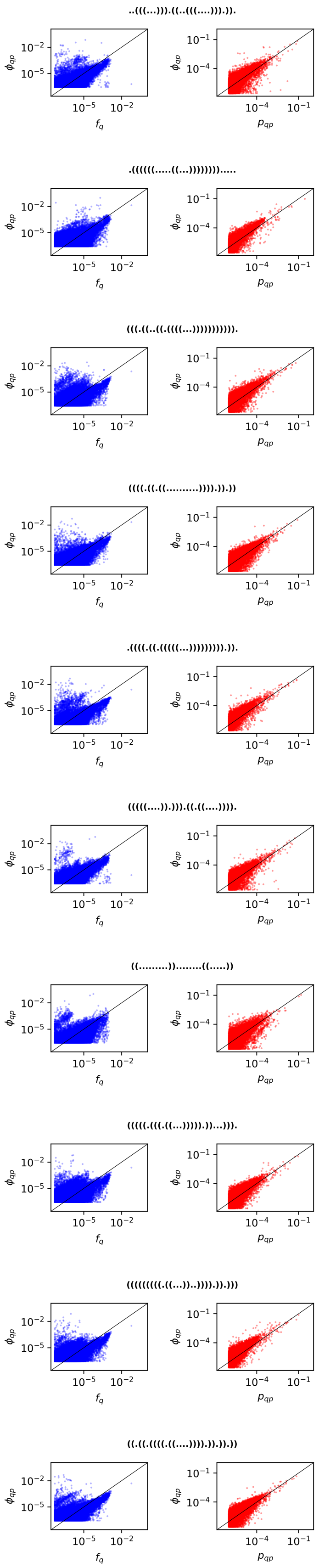

Figure S11: Continued from Fig S9

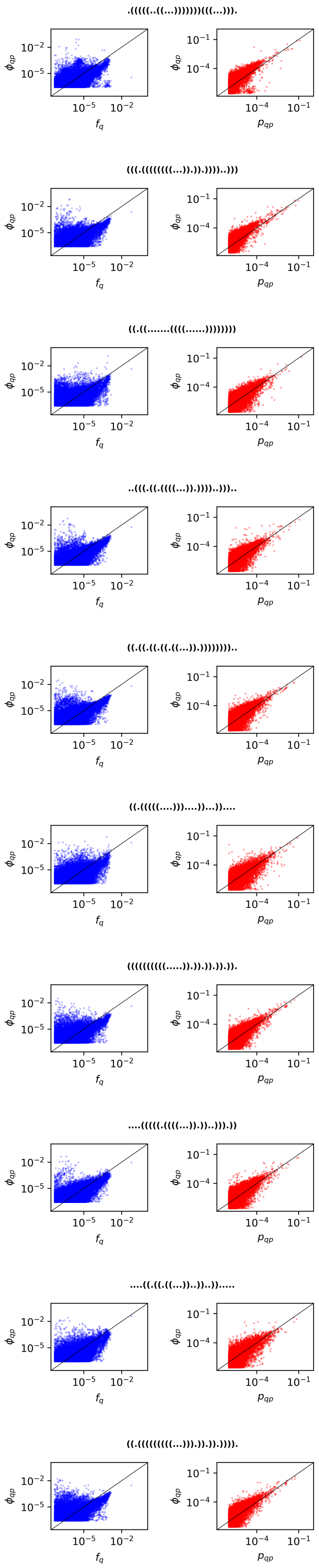

Figure S12: Continued from Fig S9

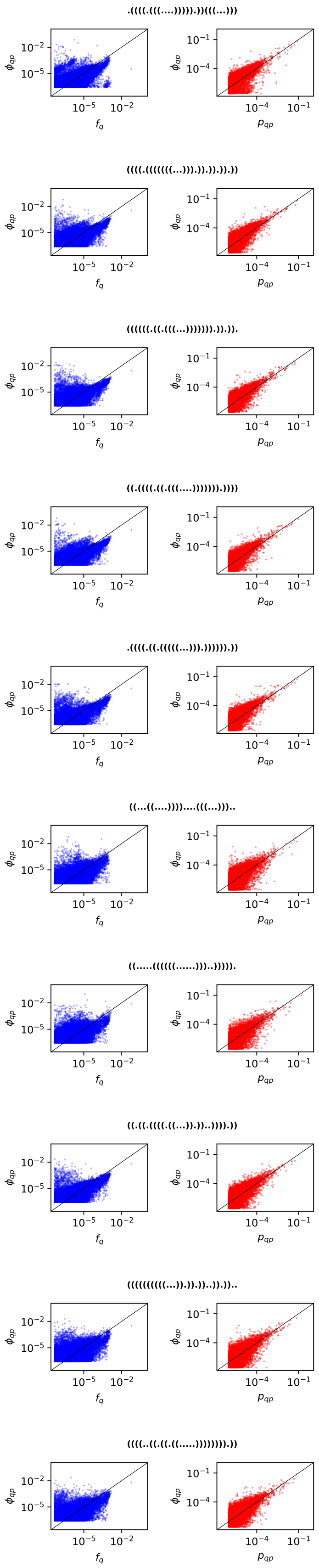

Figure S13: Continued from Fig S9

S3.2 HP protein model

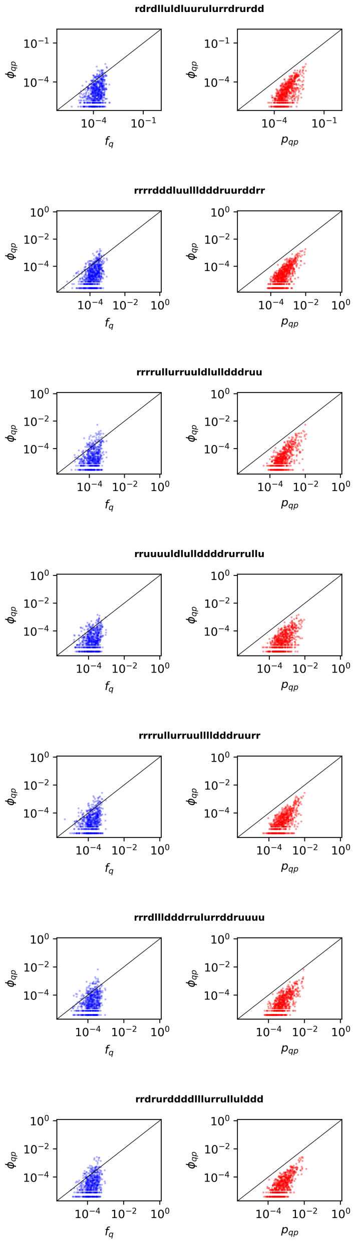

Figure S14: ***Further data for different initial HP structures*** *p: each initial structure p is shown in a separate row, with the structure given in the row title. Here we use the following convention for representing 2D lattice structures as strings: we walk along the structure and record "u" for a step upwards on the grid, "d" for a step down and "r/l" for right or left. Otherwise, the figure has exactly the same format as Figs. 3D & E in the main text. Continued in Figs S15-S18. The structures are selected in evenly spaced intervals from a full list of HP structures, ranked in descending order by phenotypic frequency, such that both frequent and rare structures appear in the plot, and the same structures will be used in Figs S24-S26 & S30 below.*

Similarly for the HP model, we show additional data for 35 different choices of the initial structure p. These 35 structures are selected in evenly spaced intervals from a full list of HP structures that is sorted by neutral set size, such that again we have a range of structures with a range of neutral set sizes (and thus thermodynamic stabilities and mutational robustness values since these are correlated [5, 12]). The results are shown in Figs S14-S18 and support

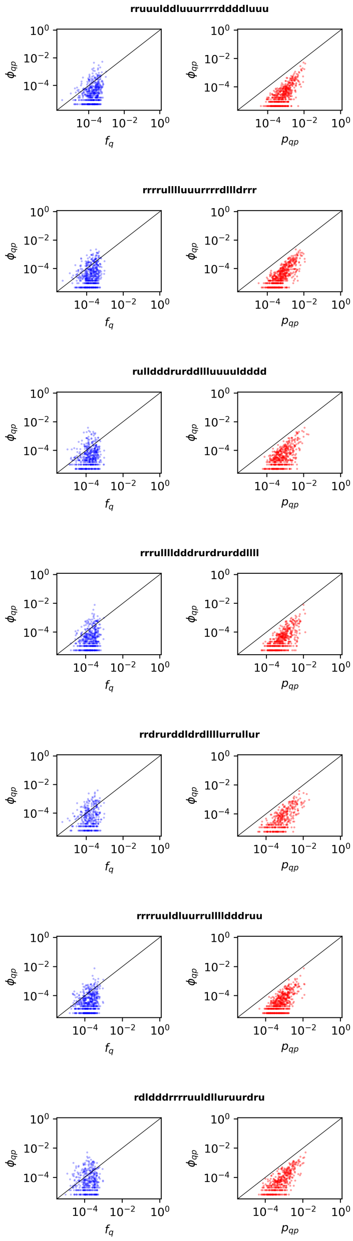

Figure S15: Continued from Fig. S14.

the conclusions from the main text: the biophysical approach (left column) is a much better indicator of p_{qp} differences than phenotypic frequencies (right column).

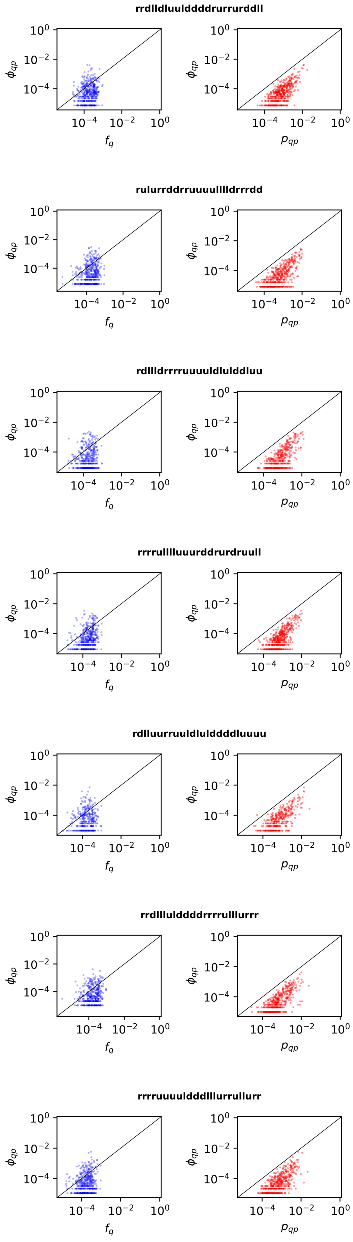

Figure S16: Continued from Fig. S14.

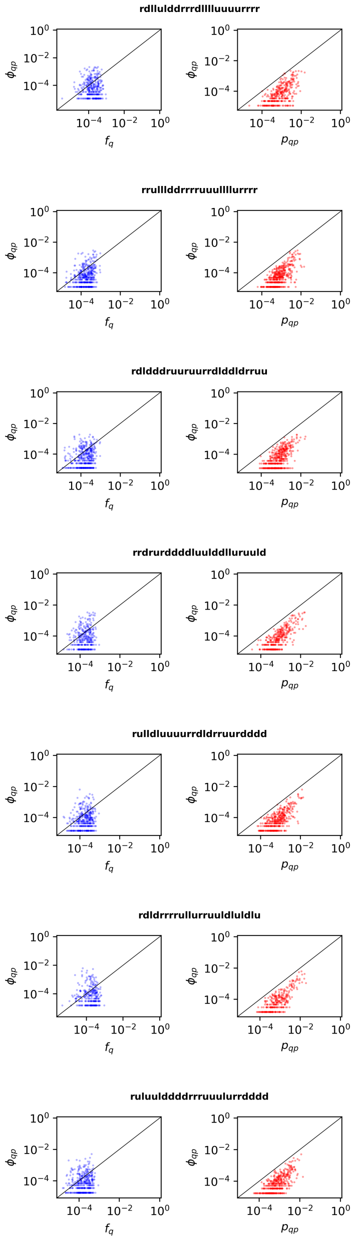

Figure S17: Continued from Fig. S14.

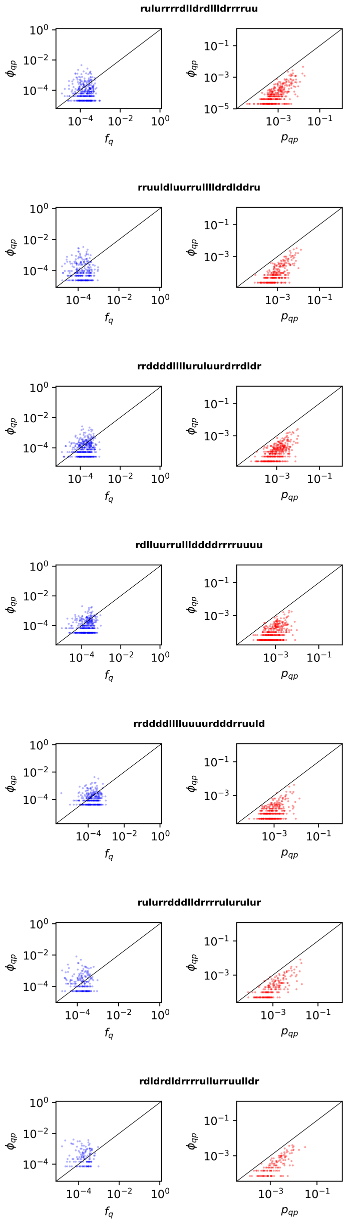

Figure S18: Continued from Fig. S14.

S4 Alternative approach: structural similarity as an indicator of mutation probabilities

So far, we have shown that the Boltzmann-ensemble-based biophysical quantity p_{qp} is a better indicator of phenotype mutation probabilities than phenotypic frequencies f_q. In this section, we test a few further quantities that might be accurate indicators of mutation probabilities. All of these quantities formalise the intuition that small structural changes should be easier to achieve through mutations than transitions between entirely different structures (see for example ref [5, 13]). Since the concept of similarity can be quantified in a variety of ways, we test a few different quantities in the following sections. We find that none of them is as good an indicator of phenotype mutation probabilities as the biophysical quantity p_{qp}.

S4.1 RNA secondary structures

S4.1.1 RNA structural distance quantified by the base pair distance

The simplest way of capturing the structural difference between two RNA secondary structures is to use the base pair distance between p and q, i.e. the number of base pairs that are either in p and not in q or vice versa. This established structural distance is implemented in the ViennaRNA package [8]. In Fig S19 we plot this distance against the mutation probability ϕ_{qp} from p to q, to test if it might be an indicator of ϕ_{qp} differences. We find that base pair distance behaves more like an upper bound: structural transitions with high ϕ_{qp} tend to have low base pair distances, but there are also examples of structural transitions with low ϕ_{qp} values that still have low base pair distances. Biophysically, this can be interpreted as follows (loosely following Fontana and Schuster [9]): for example, adding two base pairs as an extension to an existing stack is energetically much more favourable than adding two base pairs as a new stack (see ref [7] for approximate calculations), and thus we would expect that mutations are more likely to achieve the former structural change than the latter, even though the same number of base pairs is added in both structural changes and thus the base pair distance is the same.
There is another argument that explains why ϕ_{qp} values cannot be approximated well: the base pair distance is the same, regardless of whether we compare p to q or q to p, but ϕ_{qp} and ϕ_{pq} can differ by several orders of magnitude if the neutral set sizes of p and q differ since it is easier to access a large neutral set from a small one than vice versa [15].
One way of understanding, why our Boltzmann-based approach is better at approximating ϕ_{qp} trends goes as follows: likely structural transitions tend to be to structures that are structurally similar to the initial structure, but not *all* structures that are structurally similar have high transition probabilities. Similarly, Boltzmann states close to the mfe structure tend to be structurally similar to the mfe structure [16], but presumably not all Boltzmann states that are structurally similar to the mfe structure are among the energetically lowest-lying states.

S4.1.2 RNA structural distance quantified in terms of shared compatible sequences

For completeness, we repeat out analysis with similarity measures based on the number of shared compatible sequences between the initial structure p and the final structure q, since such an approach was suggested in ref [15], who in turn cite J. Weber's 1997 PhD thesis. “Compatible” simply means that a sequence could form the base pairs that make up a structure s, i.e. it has one of the base pairs AU, GC or GU at each paired position. A sequence is typically compatible with several structures, but only has one mfe structure. Thus, there are two ways in which the number of shared compatible sequences could be computed for our purposes: the first option is to count what fraction of sequences that are compatible with p are also compatible with q. The second option is to argue that ϕ_{qp} is defined relative to the neutral set of p, and the same logic should be applied here. Then we would count, what fraction of sequences in the neutral

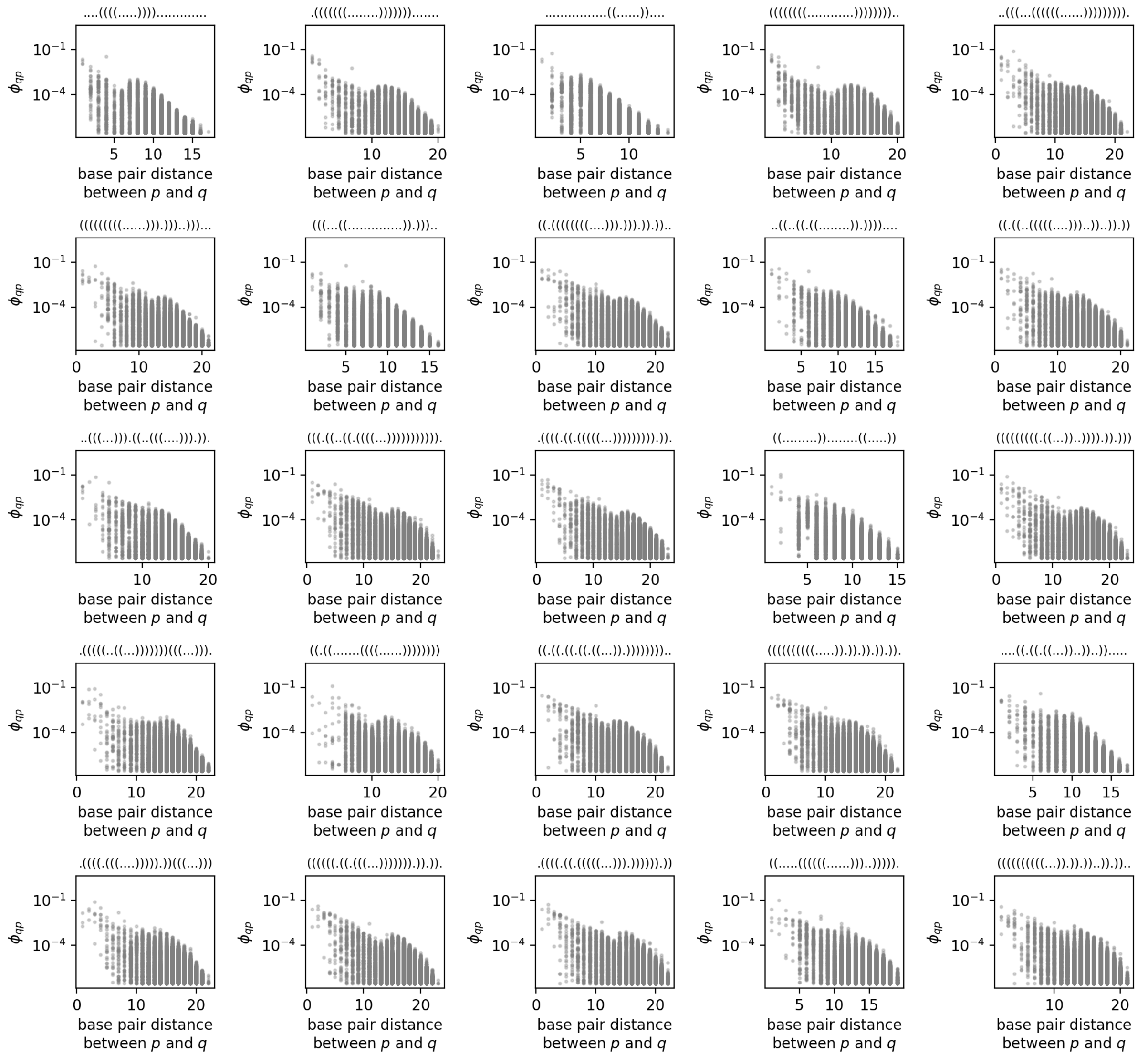

Figure S19: ***Mutational transition probability*** ϕ_{qp} ***versus structural difference between RNA structures*** *p* ***and*** *q: each subplot shows data for one fixed initial structure p, which is given in the subplot title (every second structure from a frequency-ordered list of the* 50 *structures in our dataset, the highest-frequency structures are shown first). The* ϕ_{qp} *values for structural changes to new structures q are plotted against the base pair distance between p and q, which is calculated using the ViennaRNA package [14]. We find that base pair distance behaves only like an upper bound of* ϕ_{qp} *values: all high* ϕ_{qp} *values correspond to low base pair distances, but there are also low* ϕ_{qp} *values with relatively low base pair distances.*

set of the initial structure p are compatible with the target structure q. Figs. S20 & S21 test whether either of these quantities could be an indicator of ϕ_{qp} differences. As before for base pair distances, we find that the shared number of compatible sequences can give a sort of upper bound, but not a useful indicator.

S4.1.3 RNA structural distance quantified using conditional complexities

Finally, we turn to a recent approach based on algorithmic information theory [10]. In this approach, an upper bound to $\log \phi_{qp}$ is given by the conditional complexity of the new structure q given the initial structure p. This quantity is fundamentally information theoretic in nature, but intuitively it predicts that $\log \phi_{qp}$ could be high if either the old and new structures are

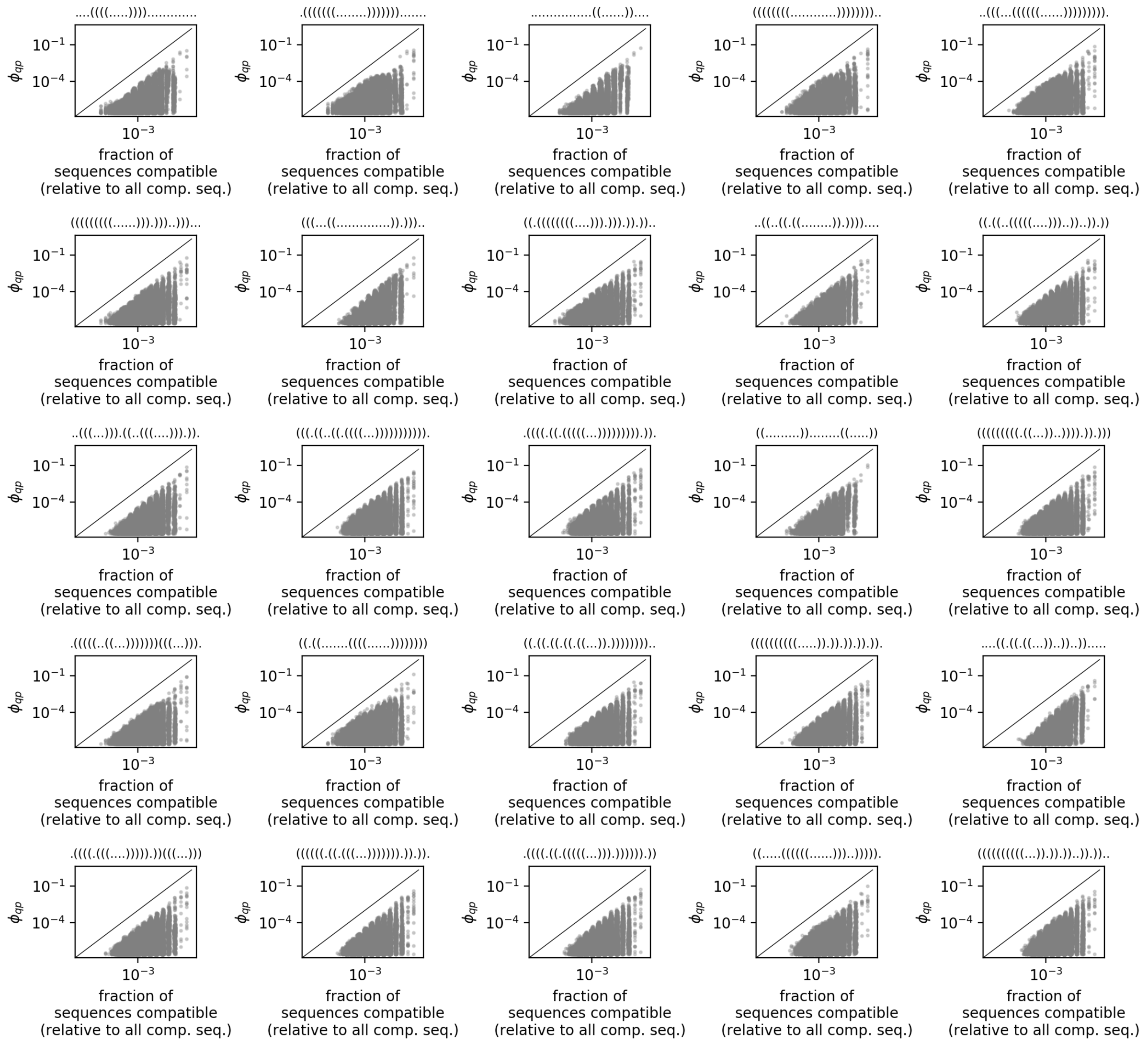

Figure S20: ***Mutational transition probability ϕ_{qp} versus overlap in the number of compatible sequences between RNA structures p and q:*** *same as Fig. S19, but here we test if compatible sequences can be an indicator of ϕ_{qp} values. Thus the data on the x-axis quantifies, what fraction of the sequences that are compatible with p, are also compatible with q. As before, this can merely give an idea of an upper bound on ϕ_{qp} values. The overlap was estimated by generating a sample of 2×10^5 random compatible sequences for each initial structure p, and testing their compatibility with the other structures q.*

highly similar or the new structure is algorithmically very simple. We compute this quantity following the methods in the original paper by Dingle et al. [10] and find that this quantity indeed gives an upper bound of ϕ_{qp} values (Fig S22), but cannot reliably tell us anything beyond an upper bound.

S4.1.4 Systematic comparison of potential ϕ_{qp} indicators

So far, we have presented a range of quantities that might be indicators of ϕ_{qp} and plotted each quantity against ϕ_{qp} values for a range of initial structures p. Here, we conclude this analysis with a systematic side-by-side comparison.

One way of quantifying the usefulness of each potential ϕ_{qp} indicator is to compute, how many of the top-30 ϕ_{qp} values for a given initial phenotype p can be identified with each potential

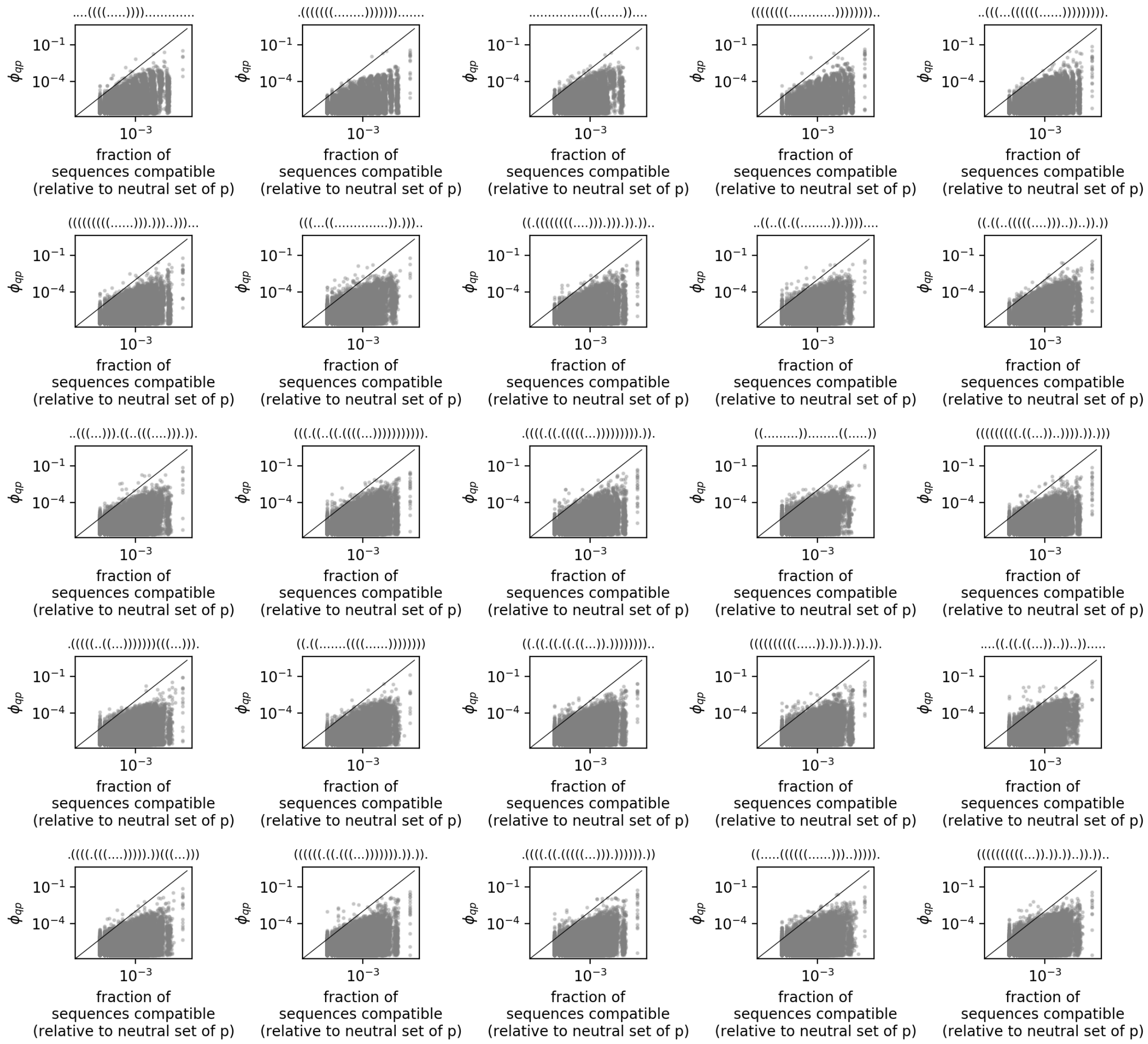

Figure S21: ***Mutational transition probability ϕ_{qp} versus overlap in the number of compatible sequences between RNA structures p and q****: same as Fig. S21, but here the overlap is evaluated relative to the neutral set of p, rather than all sequences that are compatible with p. As before, we can merely give an idea of an upper bound on ϕ_{qp} values. The overlap in the number of compatible sequences was estimated based on the existing sequence sample of 2×10^5 sequences from the neutral set of each initial structure p.*

ϕ_{qp} indicator. This approach was used in the main text, but here it is not suitable because distance metrics like the base pair distance are discrete, and therefore there may be ties and we cannot determine uniquely, which structures are predicted to have the 30 highest values. Therefore we choose a different way to quantify how well each approach captures ϕ_{qp} values: we compute the Pearson correlation coefficient between each quantity and the ϕ_{qp} values on a log-log scale (lin-log for the AIT approach and base pair distance), to quantify the extent to which we can predict $\log \phi_{qp}$ differences with each approach. The disadvantages of this method (and the reason why it is not used in the main text) is that because of the log-log scaling, zero values cannot be included and so cases, in which $\phi_{qp} = 0$ and $f_q \gg 0$ are not accounted for. In addition, structures with low ϕ_{qp} values and therefore large sampling errors will lead to larger artefacts in the Pearson correlation coefficients than in the method used in the main text. However, we present the Pearson correlation data in spite of these caveats for two reasons: it provides an alternative to the method used in the main text and can be applied to data from

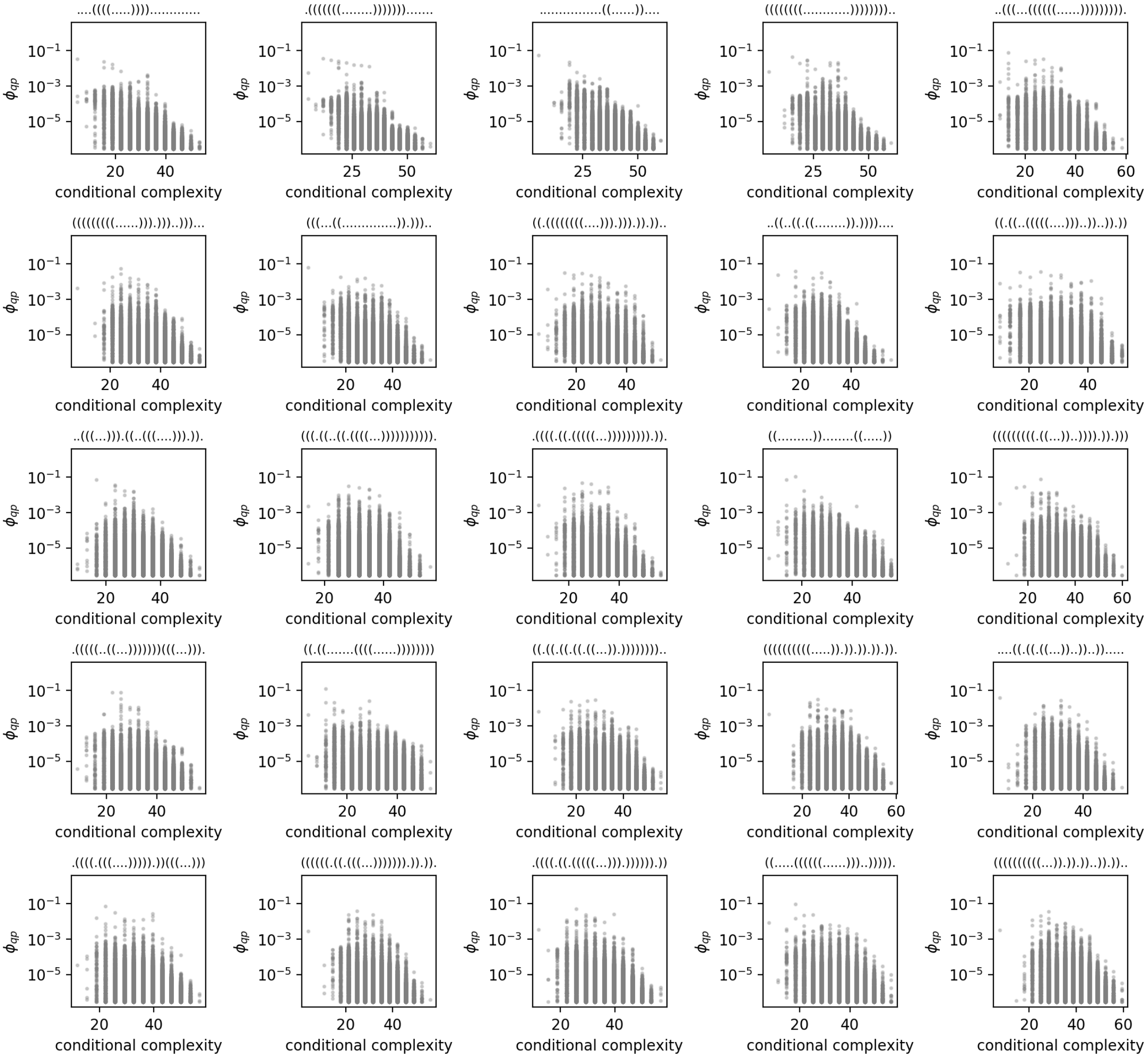

Figure S22: ***Mutational transition probability*** ϕ_{qp} ***versus conditional complexity of*** q ***given*** p ***for RNA structures****: same as Fig. S19, but here we test if conditional complexities can be an indicator of* ϕ_{qp} *values by plotting the conditional complexity of* q *given* p *on the x-axis. We find that likely phenotypic changes (i.e. high* ϕ_{qp} *values) tend to correspond to low conditional complexity values, but not vice versa: for a given conditional complexity value, we often find a range of* ϕ_{qp} *values, including likely and unlikely ones. This is consistent with the theory in ref [10], which predicts that there is a log-linear upper bound in the* ϕ_{qp}*-conditional-complexity relationship, but no lower bound.*

all approaches.

We use the following conventions for computing the correlation values: sampled values with insufficient data are treated as zeros (ϕ_{qp} and f_q values need to be based on at least five instances of q in the sample to be treated as non-zero; and estimated p_{qp} values need to be at least 10^{-6}; these are the same conditions as applied in the scatterplots). For the remaining data points, we choose a log-log scale due to the many orders of magnitude spanned by the ϕ_{qp} values (lin-log for the AIT approach and base pair distance), thus excluding zero values. The computed Pearson correlation coefficients (Fig. S23) are in agreement with what we have found so far: the biophysical Boltzmann-ensemble-based approach has the highest scores.

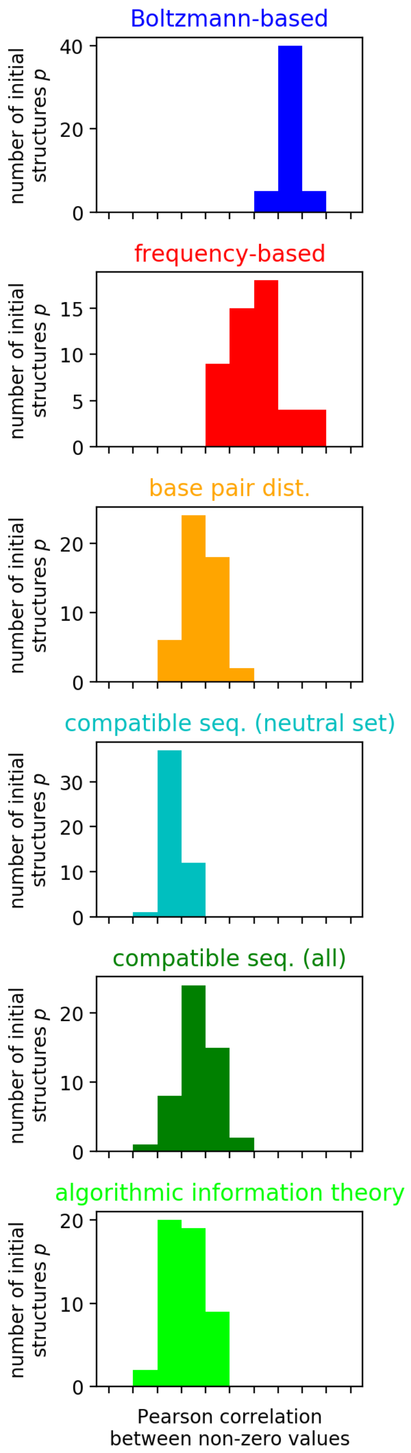

Figure S23: ***Scoring all approaches for RNA secondary structures using the Pearson correlation coefficient:*** *for each of the* 50 *initial RNA structures p for which we have data, we compute the Pearson correlation coefficient to compute, how well each potential* ϕ_{qp} *indicator captures trends in the* $\log \phi_{qp}$ *data (reporting absolute correlation coefficients since a negative correlation is as useful as a positive one): the biophysical Boltzmann-ensemble-based quantity* p_{qp} *(blue), the phenotypic frequency* f_q *(red, as in ref [5]), the base pair distance (orange, as explained in section S4.1.1), the fractional overlap in compatible sequences relative to the neutral set of p (cyan, as explained in section S4.1.2) the fractional overlap in compatible sequences relative to the compatible sequences of p (green, as explained in section S4.1.2) and the conditional complexity (lime, as in ref [10] and explained in section S4.1.3).*

S4.2 HP protein model

S4.2.1 HP structural distance quantified by the contact map overlap

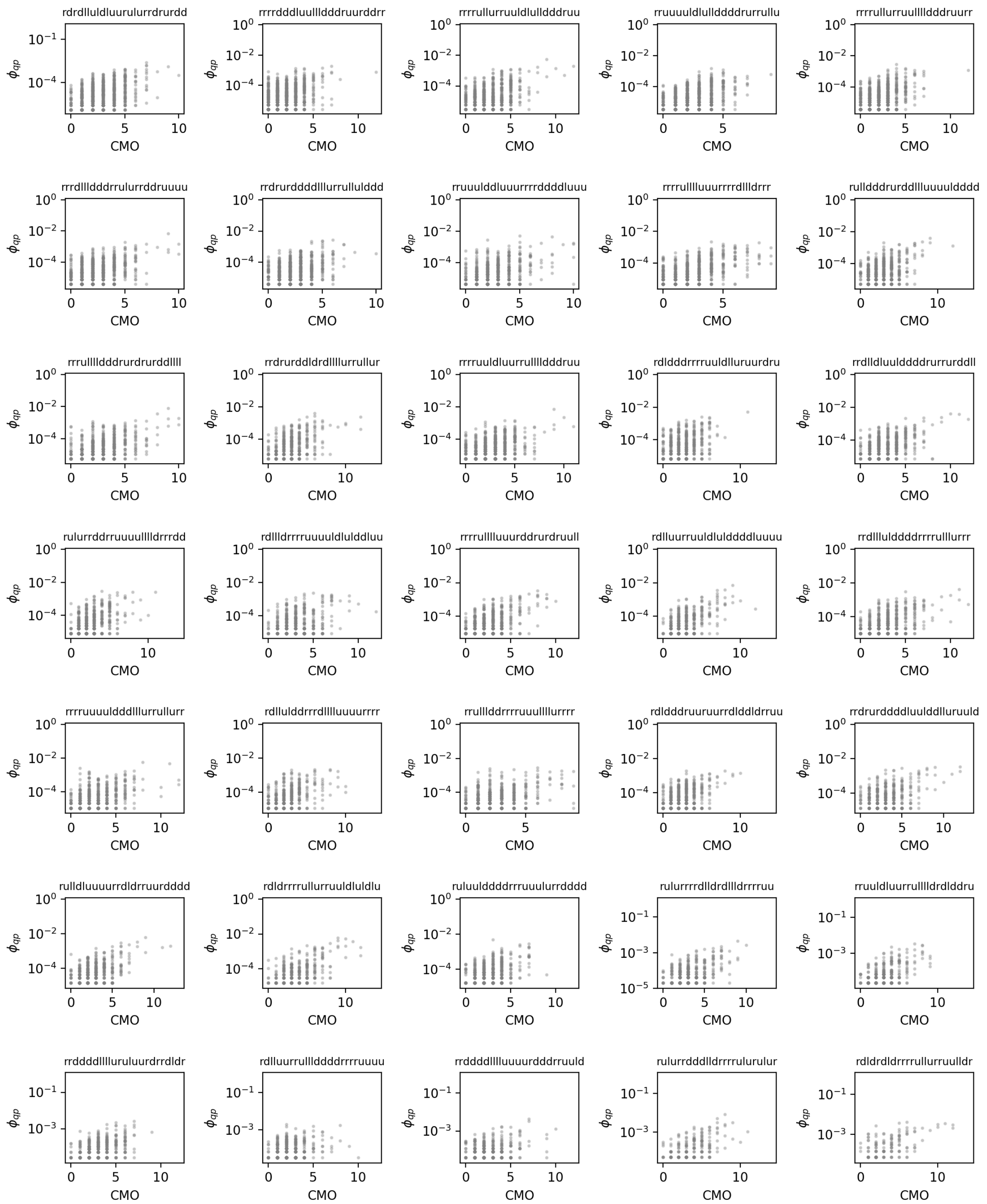

Figure S24: ***Mutational transition probability ϕ_{qp} versus structural similarity between HP protein structures p and q, measured with the contact map overlap (CMO)****: each subplot shows transitions for one fixed initial structure p, which is given in the subplot title (the same set of 35 structures as in Figs. S14-S17).*

For protein structures, we also test if structural similarity between two structures p and q

could be used as an indicator for ϕ_{qp} values. One established measure of structural similarity for proteins is the contact map overlap [17] between two structures p and q, which counts, how many of the spatially adjacent amino acids in structure p are also adjacent in structure q. In Fig. S24, we investigate whether this measure of protein structural similarity could be an indicator of mutation probabilities ϕ_{qp}. We find a weaker correspondence than for the biophysical quantity p_{qp}.

S4.2.2 HP structural distance quantified by the difference in core/surface profiles

Another way of quantifying the similarity between two HP model protein structures is to evaluate how many of the sequence positions that are in the core of structure p are also located in the core of the second structure q. This might be a more biophysically relevant measure of structural similarity since the core-surface profile is a structural quantity that is especially important for the GP map [18]. However, the data (Fig. S24) does not show a strong correspondence between this measure of structural similarity and the mutation probabilities ϕ_{qp}.

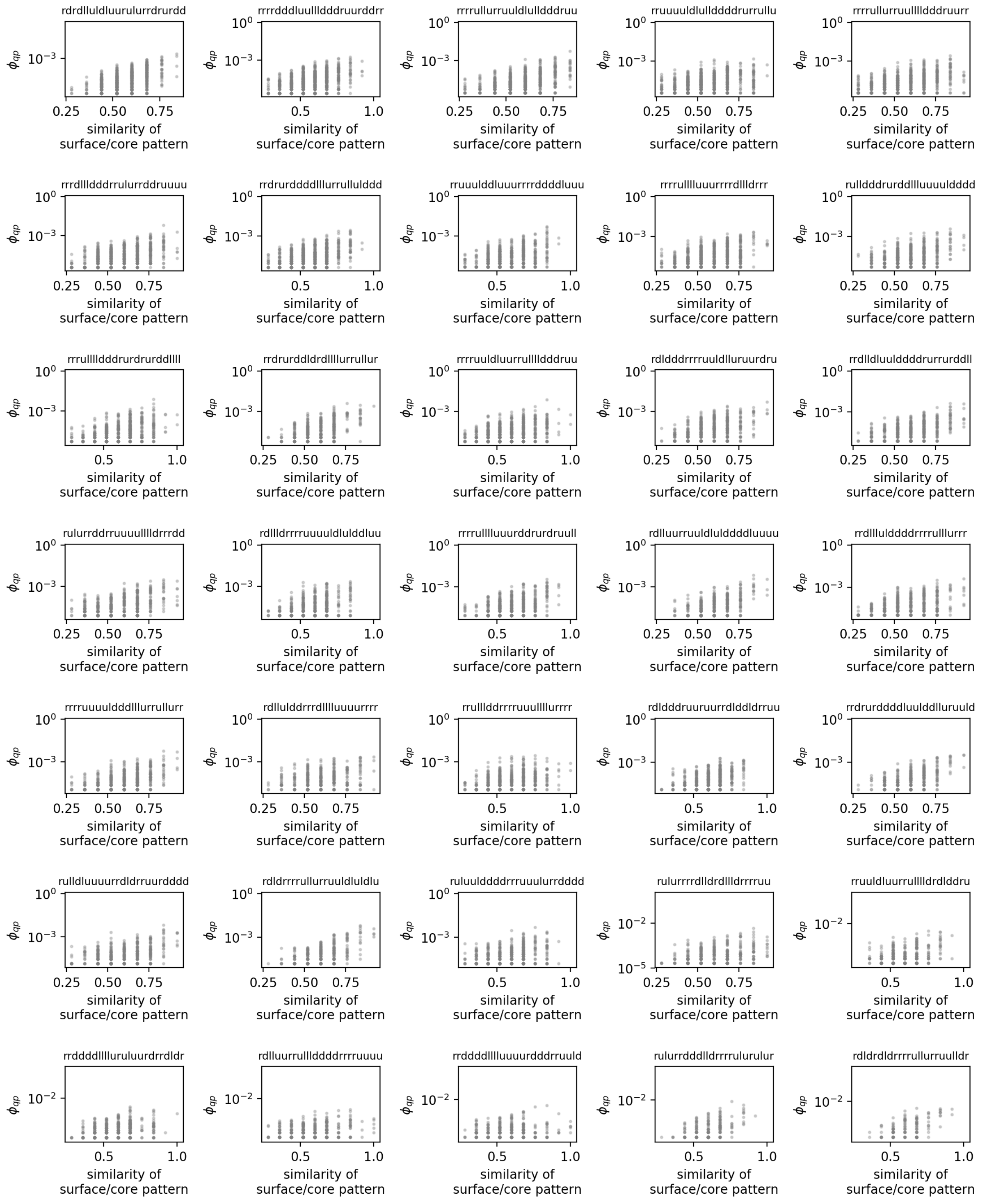

Figure S25: ***Mutational transition probability*** ϕ_{qp} ***versus structural similarity between HP protein structures*** p ***and*** q*: same as Fig S24, but here the similarity is quantified by the similarity of their surface/core patterns, i.e. the fraction of sequence positions that are either in the core of both structures* p *and* q *or on the surface of both structures.*

S4.2.3 HP structural distance quantified using conditional complexities

Finally, we also apply the recent approach based on algorithmic information theory [10], which should predict an upper bound on mutation probabilities ϕ_{qp} using conditional complexities.

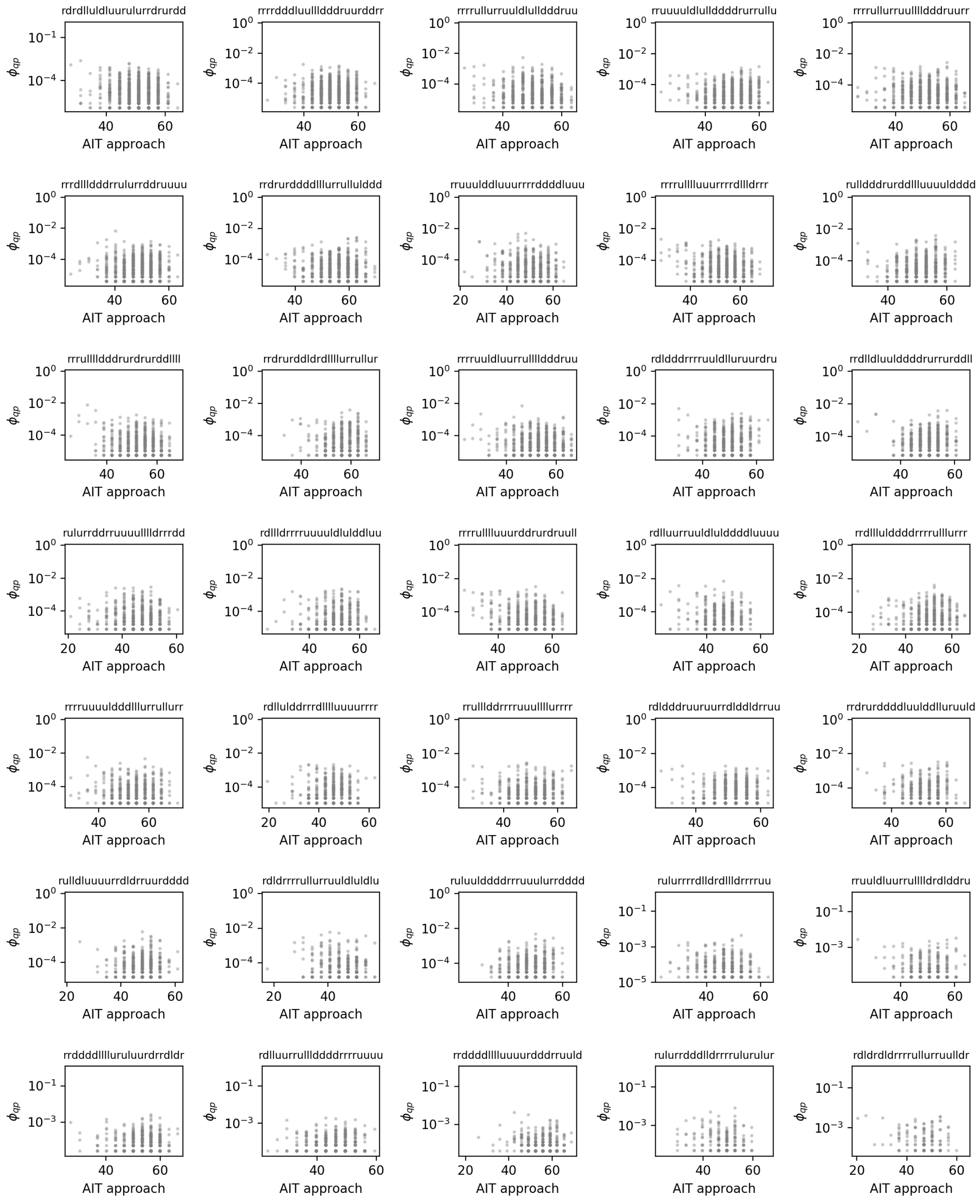

Figure S26: ***Mutational transition probability ϕ_{qp} versus conditional complexity of q given p for HP protein structures****: same as Fig S24, but here the data on the x-axis represents the conditional complexity of q given p. We do not find clear evidence for the log-linear upper bound postulated in ref [10].*

For the HP protein model, there is currently no established structural representation for the conditional complexity calculations (as far as we are aware). Here, we simply convert the description used in the subplot titles into a binary string using a single mapping ('00' for 'right', '01' for 'down', '10' for 'left', '11' for 'up'). When we plot the relationship between

the resulting computed conditional complexity values and the mutation probabilities ϕ_{qp}, we do not even observe the predicted upper bound. Thus, conditional complexities are not a good indicator of mutation probabilities in the HP model, at least not in this representation.

S4.2.4 Systematic comparison of potential ϕ_{qp} indicators

As before for RNA structures, we have presented a range of quantities that might be indicators of ϕ_{qp} in the HP model and plotted each quantity against ϕ_{qp} values for a range of initial structures p. Here, we conclude this analysis with a systematic side-by-side comparison.
As before in section S4.1.4 for RNA, we compare the performance of each approach with Pearson correlation coefficients (Fig. S27). We find that the biophysical Boltzmann-ensemble-based quantity p_{qp} has the highest score. A second finding is that structural similarity (contact map overlap or surface/core pattern) is a better indicator of ϕ_{qp} values than phenotypic frequencies in the HP model (but not as good as the biophysical Boltzmann-ensemble-based quantity).

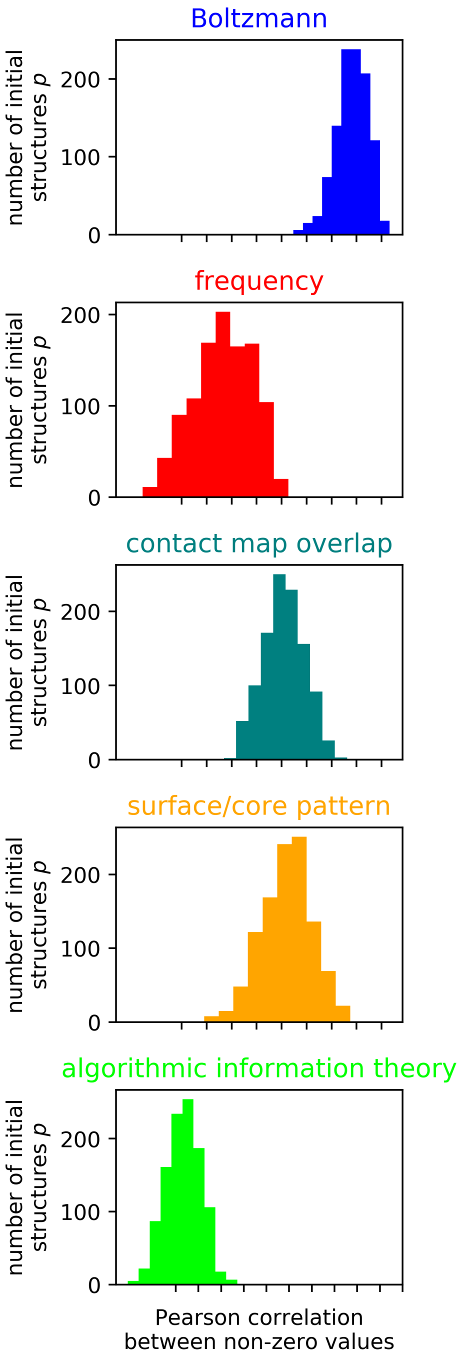

Figure S27: ***Scoring all approaches for HP protein structures using the Pearson correlation coefficient:*** *for each of the* 1081 *possible initial structures p, we compute Pearson correlation coefficients to compute, how well each potential ϕ_{qp} indicator captures trends in the* $\log \phi_{qp}$ *data (reporting correlation coefficients only since a negative correlation is as useful as a positive one): the biophysical Boltzmann-ensemble-based quantity p_{qp} (blue), the phenotypic frequency f_q (red, as in ref [5]), the contact map overlap (teal, as explained in section S4.2.1), the similarity of the core/surface patterns of structures p and q (orange, as explained in section S4.2.2) and the conditional complexity (lime, as in ref [10] and explained in section S4.2.3).*

S5 Average Boltzmann frequencies p_q and phenotypic frequencies f_q - without mfe structures

In the main text, we found that the average Boltzmann probability p_q of a structure q over a set of random sequences is correlated with the phenotypic frequency f_q of this structure (as in

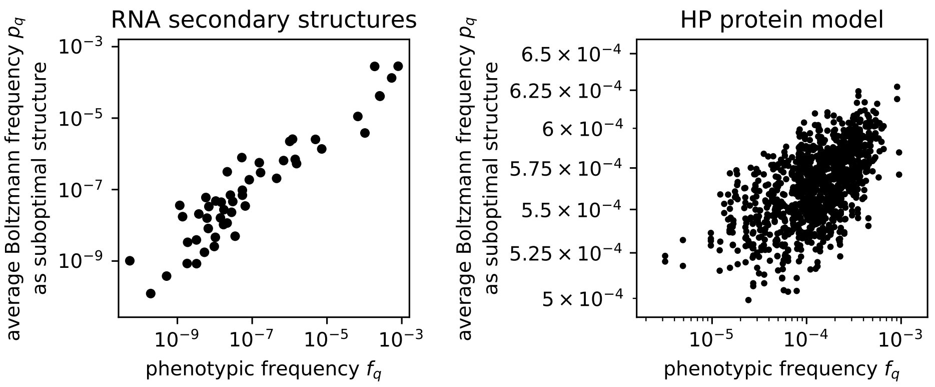

Figure S28: ***Boltzmann frequencies and phenotypic frequencies:*** *(left) the average Boltzmann frequency p_q of each RNA secondary structure q is estimated from a sample of random sequences - here a sequence is only included in the calculation if q is not the mfe structure or one of several degenerate mfe structures for this sequence. This quantity is plotted against the phenotypic frequency f_q of this structure, i.e. the probability that a random sequence folds into q as the unique mfe structure. (right) same for the HP protein model.*

ref [19, 20]). One trivial explanation for this finding could be the following: if the Boltzmann distribution of each sequence was dominated by the mfe structure, then the average Boltzmann probability p_q would simply reflect how often structure q is the mfe structure, which is exactly the definition of the phenotypic frequency f_q. To see if this trivial relationship is the reason for our observed correlation, we repeated an adjusted version of the analysis without this potential confounding factor (Fig. S28): we computed the average Boltzmann probability of a structure q over a set of random sequences but excluded sequences where it was the mfe structure or one of several degenerate mfe structures. We still find a correlation between the average Boltzmann probability of q and its phenotypic frequency for both RNA structures and the HP protein model (albeit only a weak one for the HP model). This indicates that our observed correlation is not just due to the trivial mechanism that we have just outlined.

S6 Sequence diversity in neutral sets

To get an understanding of how averages over random sequences (such as p_q) relate to averages over neutral sets (such as p_{qp}), we analyse, how the diversity of sequences in a neutral set differs from the diversity of random sequences. This analysis has already been performed for RNAshapes in our previous work [2], and further related quantities had been discussed in the past (for example [21–23]), but here we quantify this diversity systematically for the sequence lengths and models used in this analysis. For RNA (Fig. S29), we find that in large neutral sets, the distribution of sequence similarities is almost the same as the distribution among random sequences from different neutral sets, as previously found for the RNAshapes model [2]. In smaller neutral sets, sequences tend to have a higher similarity and thus lower Hamming distance. However, in all the neutral sets in Fig S29, the typical Hamming distance is still > 17, which is of the same order of magnitude as the Hamming distance between two random sequences (22.5). For HP proteins (Fig. S30), we find that two sequences from the same neutral set are more similar than two random sequences, but again there is some diversity within a neutral set: in the neutral sets shown here, two sequences typically have a Hamming distance between seven and eleven, compared to 12.5 between two random HP sequences.

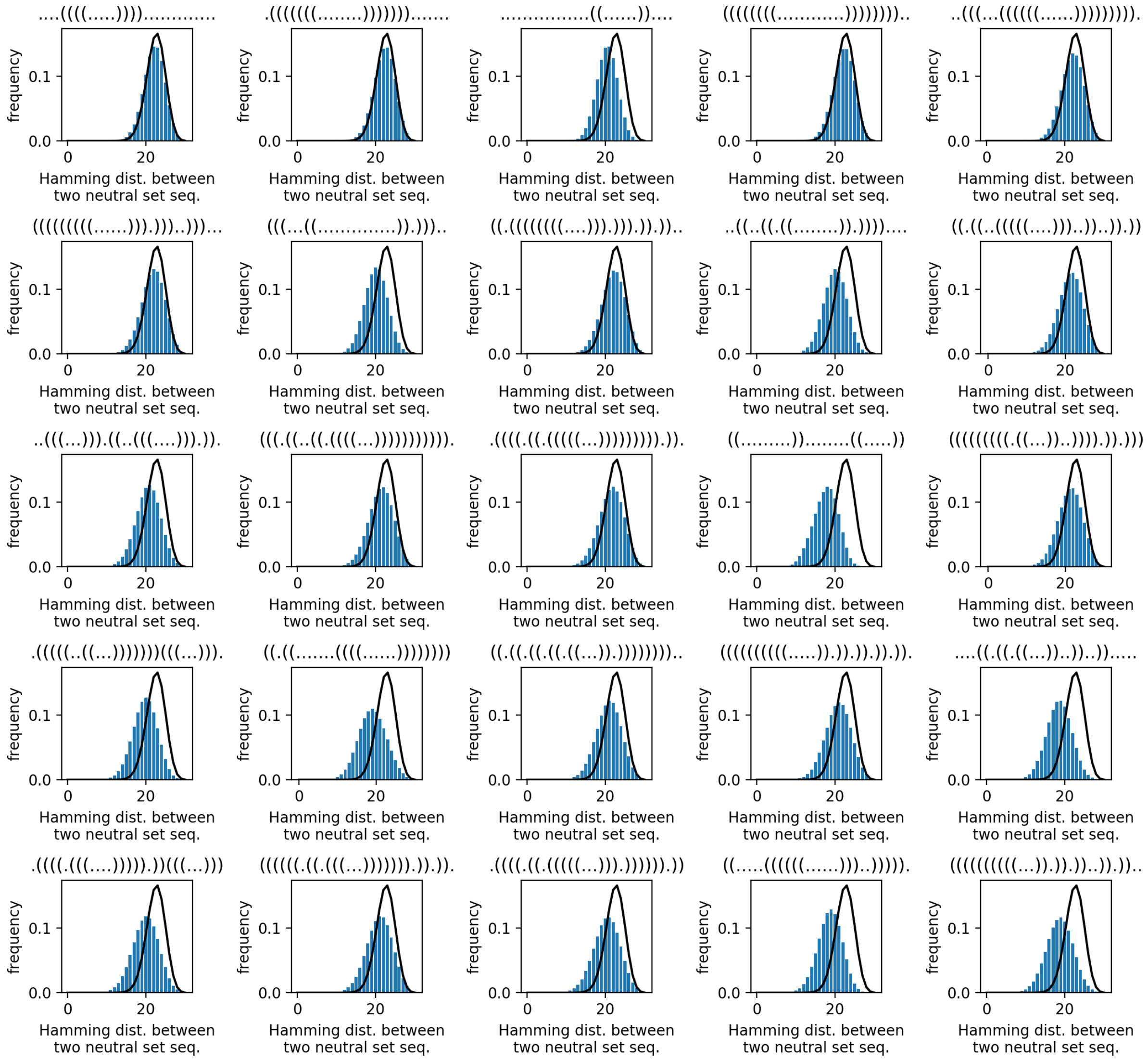

Figure S29: ***Diversity of sequences within several RNA neutral sets:*** *each plot focuses on the neutral set of one structure p (using the same selection as the RNA figures above). For each neutral set, we plot the Hamming distance distribution between different sequences in the selected neutral set (blue histogram), together with the Hamming distance distribution of random sequences (black line), as in our previous work on RNAshapes [2]. This data is based on* 10^4 *sequence per neutral set taken from the sequence samples from the* p_{qp} *calculations.*

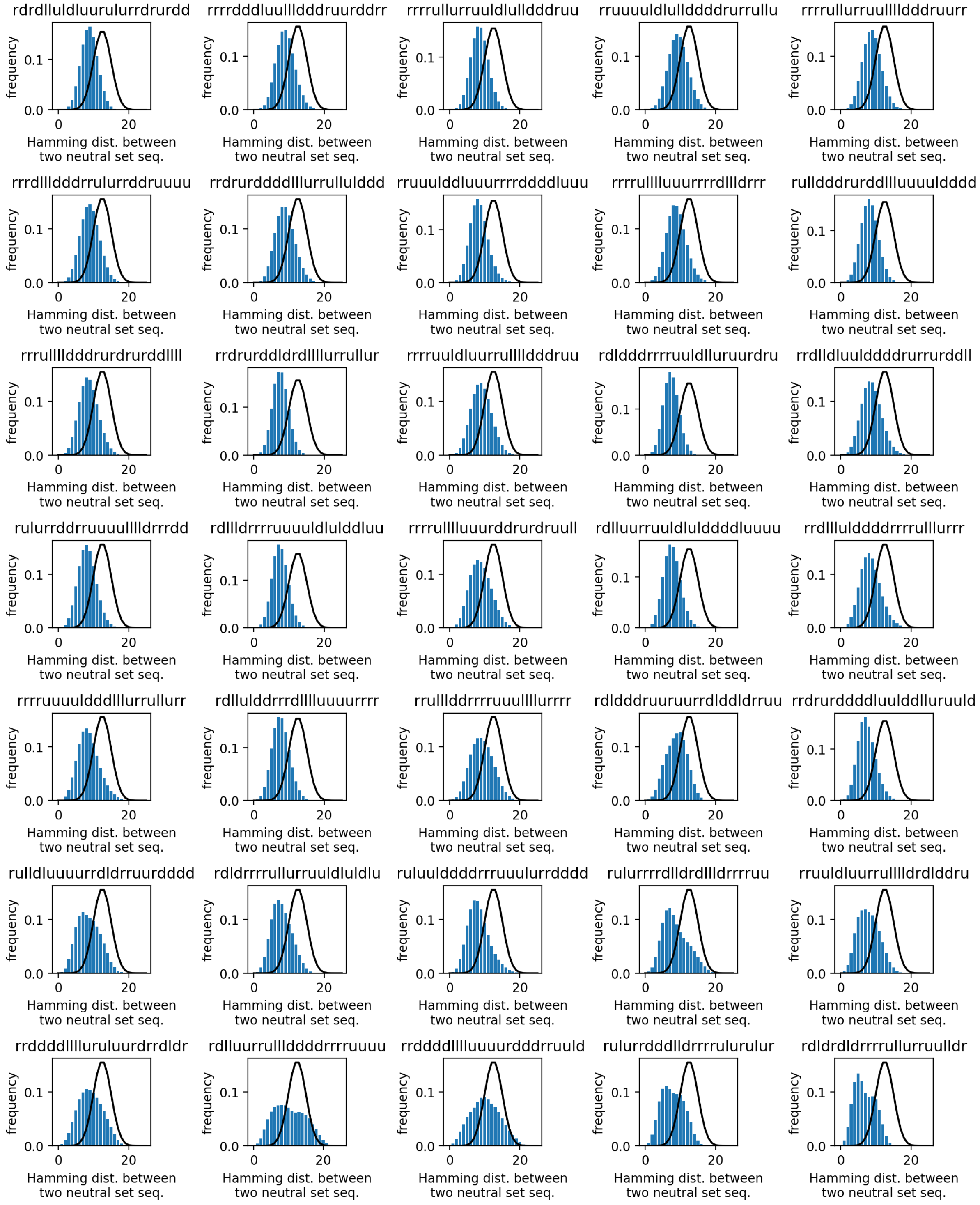

Figure S30: ***Diversity of sequences within several HP neutral sets:*** *each plot focuses on the neutral set of one initial structure p (using the same selection as the HP figures above). For each neutral set, we plot the Hamming distance distribution between different sequences in our neutral set (blue histogram), together with the Hamming distance distribution of random sequences (black line). This data is based on* 10^3 *sequences chosen randomly with replacement from each neutral set.*

S7 Different reduced temperature parameters in the HP model

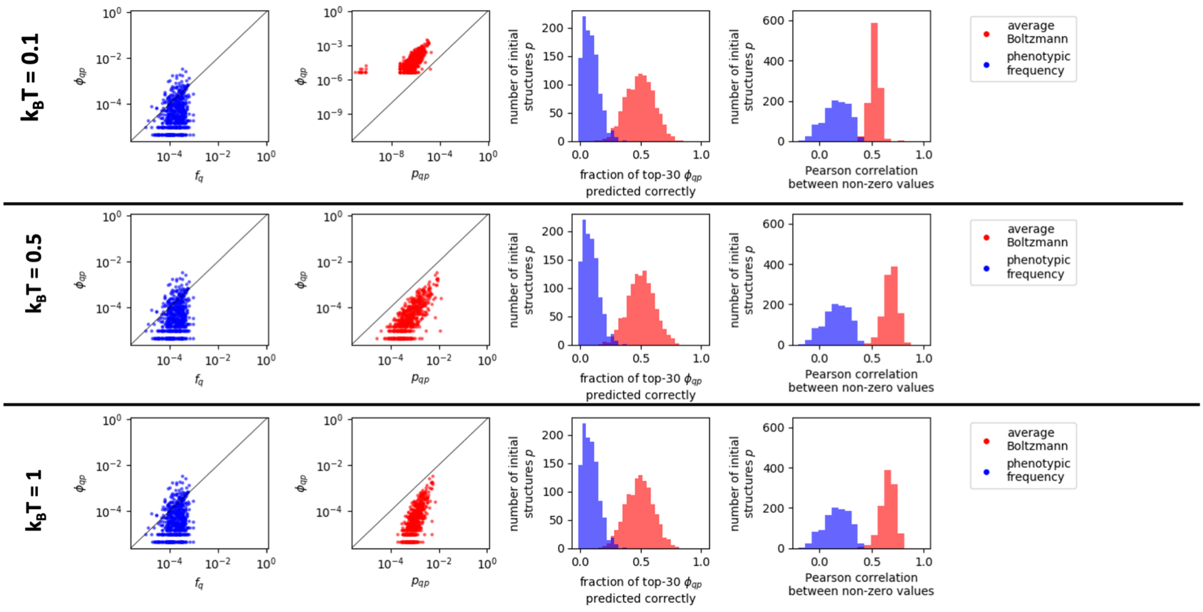

Figure S31: ***Comparison of the frequency-based and biophysical Boltzmann-ensemble-based hypotheses for HP protein structures at different reduced temperatures:*** *this plot is a repetition of the analysis in the main text (Fig. 3D-G), but for three different reduced temperatures, one in each row -* $k_BT = 0.1$*,* $k_BT = 0.5$ *and* $k_BT = 1$*. The structure p used as an example in the first two columns is the same as in the main text. Note that neither* f_q *nor* ϕ_{qp} *depend on the reduced temperature, only* p_{qp}*, so the 'blue' data for the frequency-based hypothesis are the same in all three rows, and just included as a reference.*

For RNA, the energy model is based on measured parameters that apply at a default temperature of $T = 37°$ [24], and thus this is the default temperature for ViennaRNA structure predictions. This means that the temperature we use to calculate Boltzmann weights w_s in the expression $w_s \propto \exp(-G_s/(k_BT))$ should be set to $T = 37°$ for consistency.
The HP protein model is more abstract, the free energy values are dimensionless and thus such a direct link to physical temperatures does not exist. Thus, we need to set a reduced temperature relative to the dimensionless contact energy parameters between H and P residues ($E_{HH} = -1$ and $E_{HP} = E_{PH} = E_{PP} = 0$). In ref [25], a reduced temperature of $k_BT = 1$ was used. We investigate three reduced temperatures: $k_BT = 0.1$, $k_BT = 0.5$ and $k_BT = 1$. We find that likely phenotypic transitions are well-captured by the biophysical Boltzmann-frequency-based approach for all three reduced temperatures (Fig. S31). In addition, we find that the correlation between the average Boltzmann frequency p_q of a structure q and its phenotypic frequency f_q holds in all cases, whether minimum-free-energy (mfe) structures are included or not (columns A and B in Fig. S32). Thus, our qualitative results are robust to changes in the reduced temperature.
While our results hold for all three reduced temperatures, the data for different reduced temperatures shows two qualitative differences: First, the strength of the correlations differs for different temperatures. This is what we might expect since there are no entropic terms in the model, and so the Boltzmann frequencies change with temperature (with equal Boltzmann frequencies for all structures as $T \to \infty$), whereas the mfe structures have no temperature dependence. Thus, quantities like f_q and ϕ_{qp}, which only depend on mfe structures, are unaffected

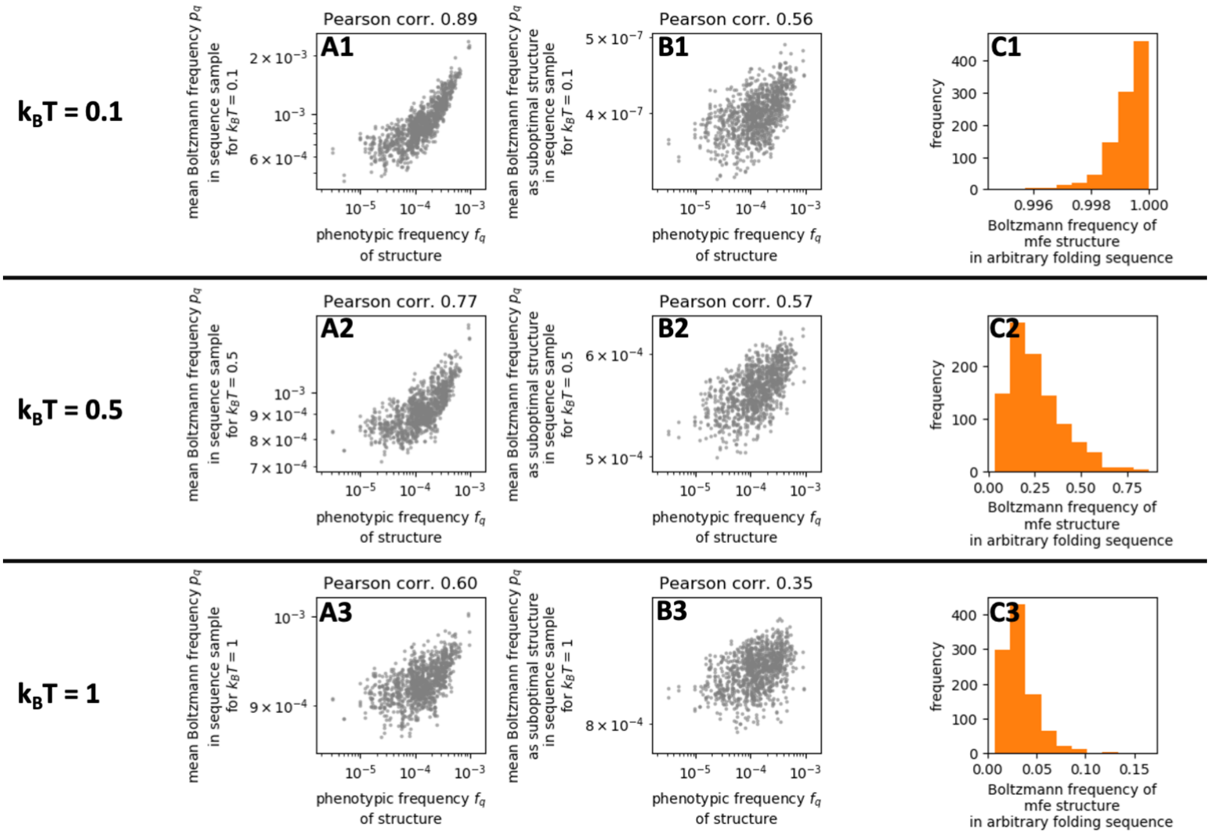

Figure S32: ***Average Boltzmann frequencies p_q for structure q and typical Boltzmann frequencies at different reduced temperatures:*** *each row uses a different reduced temperature: 1) $k_BT = 0.1$, 2) $k_BT = 0.5$ and 3) $k_BT = 1$. In each row, column A shows the relationship between the average Boltzmann frequency p_q of structure q and the phenotypic frequency f_q of that structure. We find a positive correlation in all three cases, but this becomes weaker as high-energy states are populated more evenly at higher temperature. Column B shows the same data, except that sequences where the given structure is the mfe structure are excluded from the p_q calculations, as in Fig S28. Column C quantifies how dominant the mfe structure is for a typical sequence: the histograms show the distribution of the Boltzmann frequency of the mfe structure F_{mfe} for 10^3 random sequences that fold into a unique mfe structure.*

by temperature changes. Secondly (column C of Fig. S32), we note that for $k_BT = 0.1$, a typical folding sequence folds into its mfe structure with a Boltzmann probability of $\gtrapprox 0.998$, whereas for $k_BT = 1$, this decreases to $\lessapprox 0.05$. Therefore, we use a value of $k_BT = 0.5$ throughout the main text, so that a typical sequence folds primarily into its mfe structure but has some plasticity that corresponds to protein dynamics or even fold-switching as found in ref [26].

S8 Outline of potential applications in sampling techniques

The purpose of the present paper is to understand the link between average Boltzmann ensembles and mutation probabilities and test it systematically against different predictors of mutation probabilities. This link between average Boltzmann ensembles and mutation probabilities is important in itself: first, it gives a biophysical intuition for the strong bias in mutation probabilities. As we showed, this biophysical reasoning combines the intuitions that phenotypic transitions are likely if the target structure q has a high phenotypic frequency and if it is structurally/energetically similar to the initial structure p. Secondly, the link between average

Boltzmann ensembles and mutation probabilities may be important in evolutionary processes and is therefore important for interpreting data from evolved sequences and structures. Despite this descriptive focus of the present paper, one potential application lies in sampling approaches. In the following, we will sketch some potential avenues for future work in this direction.

In models where the Boltzmann ensemble can be predicted as quickly as the mfe structure: in some models, for example the HP model, obtaining the entire Boltzmann ensembles of n sampled sequences has the same computational cost as obtaining only their mfe structures. Taking the full Boltzmann ensemble and not only mfe structures provides more nuanced data: for example a structure that is only a mfe structure in one of n sequences may be a low-Boltzmann-frequency state in several of n sequences and so we can obtain more data from the same number of n sequences if we take into account the entire Boltzmann ensemble. Our work on the links between f_q and p_q, as well as ϕ_{qp} and p_{qp} may be useful for designing methods that use this additional information from Boltzmann ensembles.

Estimating mutation probabilities ϕ_{qp} in the RNA model: we found that the mean Boltzmann frequency p_{qp} of structure q in the neutral set of the initial structure p is a good predictor of the mutation probability ϕ_{qp}. While p_{qp} is an average over continuous Boltzmann probabilities, it could be approximated by the (discrete) number of sequences that fold into p as their mfe structure and into q with high Boltzmann probability. It may be possible to estimate this number by generating a sample of bi-stable sequences using existing methods [27, 28] and selecting only sequences with p as the mfe structure and q as a high-Boltzmann-probability alternative. The total number of sequences that meet both conditions could then be approximated from the sequence sample, using existing methods that estimate the number of sequences (or a proxy for this number) from the properties of a sequence alignment, such as refs [29, 30].

References

[1] M. Weiß and S. E. Ahnert, "Using small samples to estimate neutral component size and robustness in the genotype–phenotype map of RNA secondary structure", J. R. Soc., Interface **17**, 20190784 (2020).

[2] N. S. Martin and S. E. Ahnert, "Insertions and deletions in the RNA sequence–structure map", J. R. Soc., Interface **18**, 20210380 (2021).

[3] T. Jörg, O. C. Martin, and A. Wagner, "Neutral network sizes of biological RNA molecules can be computed and are not atypically small", BMC Bioinf. **9**, 464 (2008).

[4] K. Dingle, S. Schaper, and A. A. Louis, "The structure of the genotype–phenotype map strongly constrains the evolution of non-coding RNA", Interface focus **5**, 20150053 (2015).

[5] S. F. Greenbury, S. Schaper, S. E. Ahnert, and A. A. Louis, "Genetic correlations greatly increase mutational robustness and can both reduce and enhance evolvability", PLoS Comput. Biol. **12**, e1004773 (2016).

[6] A. Wagner, "Robustness and evolvability: a paradox resolved", Proc. R. Soc. B **275**, 91–100 (2008).

[7] N. Martin and S. Ahnert, "Thermodynamics and neutral sets in the RNA sequence-structure map", EPL **139**, 37001 (2022).

[8] I. L. Hofacker, "RNA secondary structure analysis using the Vienna RNA Package", Curr. Protoc. Bioinf. **4**, 12.2.1–12.2.12 (2003).

[9] W. Fontana and P. Schuster, "Continuity in evolution: on the nature of transitions", Science **280**, 1451–1455 (1998).

[10] K. Dingle, J. K. Novev, S. E. Ahnert, and A. A. Louis, "Predicting phenotype transition probabilities via conditional algorithmic probability approximations", J. R. Soc., Interface **19**, 20220694 (2022).

[11]S. F. Greenbury, A. A. Louis, and S. E. Ahnert, "The structure of genotype-phenotype maps makes fitness landscapes navigable", Nature Ecology & Evolution **6**, 1742–1752 (2022).

[12]N. E. G. Buchler and R. A. Goldstein, "Surveying determinants of protein structure designability across different energy models and amino-acid alphabets: a consensus", J. Chem. Phys. **112**, 2533–2547 (2000).

[13]R. Wroe, H. S. Chan, and E. Bornberg-Bauer, "A structural model of latent evolutionary potentials underlying neutral networks in proteins", HFSP J. **1**, 79 (2007).

[14]R. Lorenz, S. H. Bernhart, C. H. Zu Siederdissen, H. Tafer, C. Flamm, P. F. Stadler, and I. L. Hofacker, "ViennaRNA Package 2.0", Algorithms Mol. Biol. **6**, 26 (2011).

[15]B. M. Stadler, P. F. Stadler, G. P. Wagner, and W. Fontana, "The topology of the possible: formal spaces underlying patterns of evolutionary change", J. Theor. Biol. **213**, 241–274 (2001).

[16]G. Steger and R. Giegerich, "14. RNA structure prediction", in *RNA Structure and Folding*, edited by D. Klostermeier and C. Hammann (De Gruyter, Berlin, Boston, 2013), pp. 335–362.

[17]A. Godzik, J. Skolnick, and A. Kolinski, "Regularities in interaction patterns of globular proteins", Protein Eng., Des. Sel. **6**, 801–810 (1993).

[18]H. Li, C. Tang, and N. S. Wingreen, "Are protein folds atypical?", Proc. Natl. Acad. Sci. **95**, 4987–4990 (1998).

[19]K. Dingle, F. Ghaddar, P. Šulc, and A. A. Louis, "Phenotype bias determines how natural RNA structures occupy the morphospace of all possible shapes", Mol. Biol. Evol. **39**, msab280 (2022).

[20]P. García-Galindo, S. E. Ahnert, and N. S. Martin, "The non-deterministic genotype–phenotype map of RNA secondary structure", J. R. Soc., Interface **20**, 20230132 (2023).

[21]E. Ferrada and A. Wagner, "A comparison of genotype-phenotype maps for RNA and proteins", Biophys. J. **102**, 1916–1925 (2012).

[22]U. Bastolla, H. E. Roman, and M. Vendruscolo, "Neutral evolution of model proteins: diffusion in sequence space and overdispersion", J. Theor. Biol. **200**, 49–64 (1999).

[23]P. Schuster, W. Fontana, P. F. Stadler, and I. L. Hofacker, "From sequences to shapes and back: a case study in RNA secondary structures", Proc. R. Soc. B **255**, 279–284 (1994).

[24]D. H. Mathews and D. H. Turner, "Prediction of RNA secondary structure by free energy minimization", Curr. Opin. Struct. Biol. **16**, 270–278 (2006).

[25]J. D. Bloom, J. J. Silberg, C. O. Wilke, D. A. Drummond, C. Adami, and F. H. Arnold, "Thermodynamic prediction of protein neutrality", Proc. Natl. Acad. Sci. **102**, 606–611 (2005).

[26]L. L. Porter and L. L. Looger, "Extant fold-switching proteins are widespread", Proc. Natl. Acad. Sci. **115**, 5968–5973 (2018).

[27]A. Taneda, "Multi-objective optimization for RNA design with multiple target secondary structures", BMC Bioinf. **16**, 280–299 (2015).

[28]S. Hammer, W. Wang, S. Will, and Y. Ponty, "Fixed-parameter tractable sampling for rna design with multiple target structures", BMC Bioinf. **20**, 209–221 (2019).

[29]J. A. García-Martín, P. Catalán, S. Manrubia, and J. A. Cuesta, "Statistical theory of phenotype abundance distributions: a test through exact enumeration of genotype spaces", EPL **123**, 28001 (2018).

[30]H. Jacquin, A. Gilson, E. Shakhnovich, S. Cocco, and R. Monasson, "Benchmarking inverse statistical approaches for protein structure and design with exactly solvable models", PLoS Comput. Biol. **12**, e1004889 (2016).
